# Brain multiomic profiling identifies tau-related transcriptomic dysregulation in Alzheimer’s disease

**DOI:** 10.1093/braincomms/fcag326

**Published:** 2026-08-27

**Authors:** Stephanie R Oatman, Zachary S Quicksall, Xue Wang, Jeremiah Bergman, Joseph S Reddy, Floor Vanelderen, Thuy Nguyen, Kimberly Malphrus, Sarah J Lincoln, Yuka A Martens, Na Zhao, Yu Yamazaki, Michael DeTure, Melissa E Murray, Chia-Chen Liu, Guojun Bu, Takahisa Kanekiyo, Dennis W Dickson, Mariet Allen, Nilüfer Ertekin-Taner

**Affiliations:** Department of Neuroscience, Mayo Clinic, Jacksonville, FL 32224, USA; Department of Quantitative Health Sciences, Mayo Clinic, Jacksonville, FL 32224, USA; Department of Quantitative Health Sciences, Mayo Clinic, Jacksonville, FL 32224, USA; Department of Neuroscience, Mayo Clinic, Jacksonville, FL 32224, USA; Department of Quantitative Health Sciences, Mayo Clinic, Jacksonville, FL 32224, USA; Department of Neuroscience, Mayo Clinic, Jacksonville, FL 32224, USA; Department of Neuroscience, Mayo Clinic, Jacksonville, FL 32224, USA; Department of Neuroscience, Mayo Clinic, Jacksonville, FL 32224, USA; Department of Neuroscience, Mayo Clinic, Jacksonville, FL 32224, USA; Department of Neuroscience, Mayo Clinic, Jacksonville, FL 32224, USA; Department of Neuroscience, Mayo Clinic, Jacksonville, FL 32224, USA; Department of Neuroscience, Mayo Clinic, Jacksonville, FL 32224, USA; Department of Neuroscience, Mayo Clinic, Jacksonville, FL 32224, USA; Department of Neuroscience, Mayo Clinic, Jacksonville, FL 32224, USA; Department of Neuroscience, Mayo Clinic, Jacksonville, FL 32224, USA; Department of Neuroscience, Mayo Clinic, Jacksonville, FL 32224, USA; Department of Neuroscience, Mayo Clinic, Jacksonville, FL 32224, USA; Department of Neuroscience, Mayo Clinic, Jacksonville, FL 32224, USA; Department of Neuroscience, Mayo Clinic, Jacksonville, FL 32224, USA; Department of Neuroscience, Mayo Clinic, Jacksonville, FL 32224, USA; Department of Neurology, Mayo Clinic, Jacksonville, FL 32224, USA

**Keywords:** transcriptome, expression, tau, amyloid, APOE

## Abstract

Identification of gene expression changes in postmortem brain tissue of Alzheimer’s disease donors compared to controls has implicated numerous biological pathways for Alzheimer’s disease pathophysiology. Nonetheless, there is still limited understanding of how gene expression dysregulation underpins specific proteinopathies core to Alzheimer’s disease. Here, we investigate brain transcriptomic changes in a well-characterized cohort of Alzheimer’s disease donors to identify genes and networks that associate with Alzheimer’s disease endophenotypes, including neuropathology measures (Braak stage, Thal phase and cerebral amyloid angiopathy score) and Alzheimer’s disease-related brain protein levels (Apolipoprotein E, Amyloid-β 40, Amyloid-β 42, tau and phospho-Tau). Bulk transcriptome measures were collected from the temporal cortex tissue of 477 Alzheimer’s disease donors. Following quality control, transcriptome-wide association studies were performed for each endophenotype. We used weighted gene co-expression network analysis to build co-expression networks and integrated transcriptome with epigenetic and genetic data from the same donors. We detected a total of 5740 Bonferroni-significant temporal cortex gene associations with Alzheimer’s disease endophenotypes, most of which were with brain tau levels. We discovered tau-associated co-expression modules enriched in known and novel Alzheimer’s disease pathways. We found that a *beneficial (or neutral)* brain biochemical state of higher total tau and lower phospho-Tau is associated with increased levels of synaptic, DNA damage/repair, nucleic acid metabolism and myelin processes. In contrast, in a detrimental state with lower total and higher phospho-Tau, there is upregulation of vascular and immune, and downregulation of mitochondrial and myelin pathways. There are brain gene expression perturbations that are associated with Alzheimer’s disease endophenotypes. While some of these associations are common across multiple endophenotypes, many are distinct for different Alzheimer’s disease-related proteins. Based on these findings, we propose a hypothetical model of *dynamic brain gene expression changes* that track with progressive Alzheimer’s disease proteostasis. These expression changes hold potential to serve as dynamic, precision biomarkers of brain Alzheimer’s disease progression. This study demonstrates the potential of integrative multi-omics and deep Alzheimer’s disease endophenotypes in well-characterized brain tissues to precisely uncover the complex biology of Alzheimer’s disease.

## Teaser

Oatman *et al.* studied how brain gene expression changes relate to levels of proteins core to Alzheimer’s disease neuropathology: amyloid-β and tau, in addition to APOE. Most changes are related to levels of tau protein; they propose a model of *dynamic brain gene expression changes* that tracks with Alzheimer’s disease proteostasis.

## Graphical Abstract

**Graphical Abstract:**
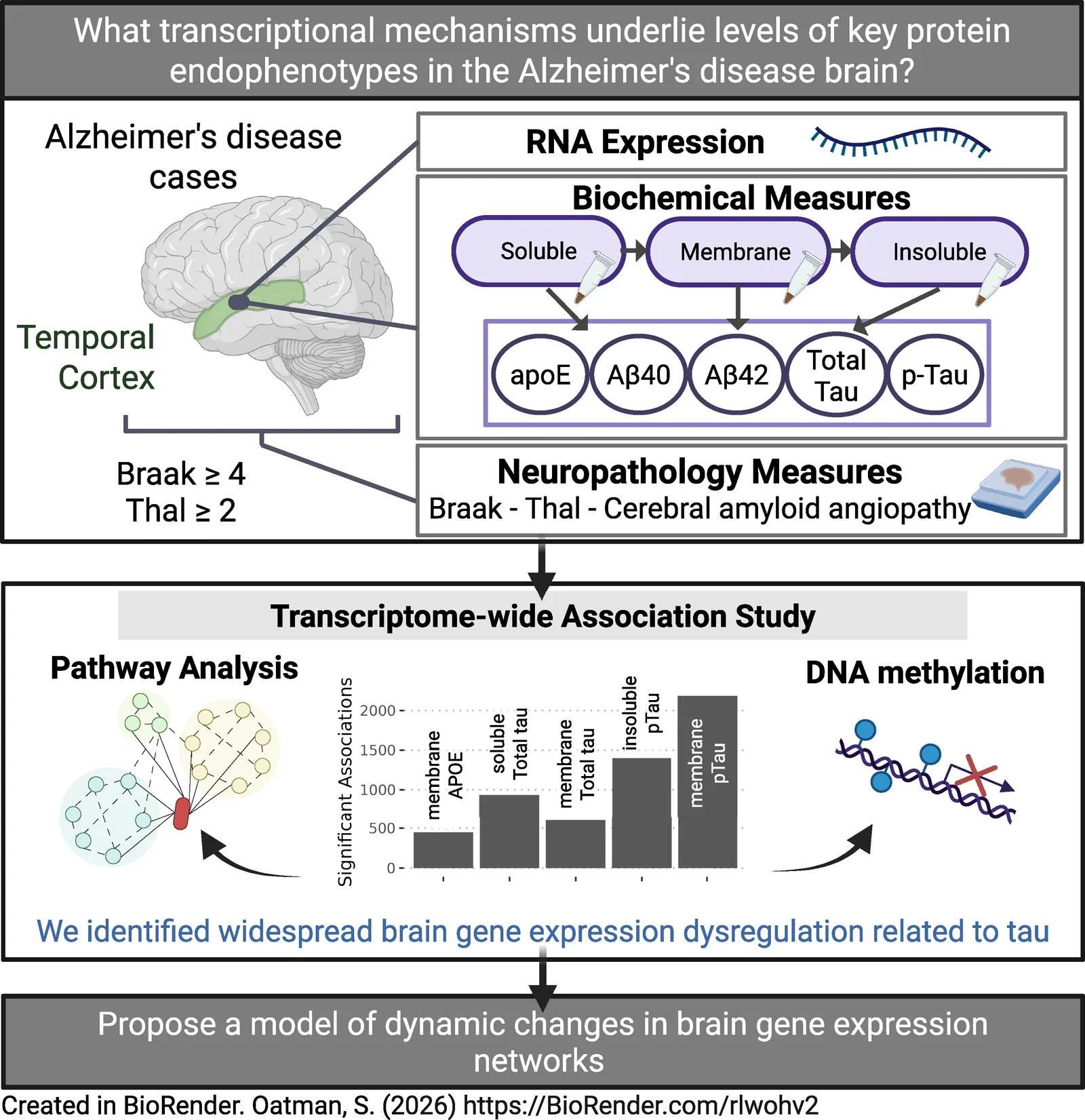

## Introduction

Alzheimer’s disease (AD) is characterized by neuropathological hallmarks of amyloid-β (Aβ) plaques and tau neurofibrillary tangles (NFTs). To date, numerous studies have identified important gene expression changes in the AD brain compared to controls, including dysregulation of pathways relating to synaptic signalling, immune responses, metabolism and others.^1-9^ Additional inclusion of multi-omic layers has further led to an increased understanding of the regulatory networks that influence these disrupted gene signalling pathways.^10,11^

Although numerous studies have characterized how levels of genes and gene co-expression networks differ between AD and non-AD donor brains,^1,2,4-8^ fewer have investigated how gene expression changes relate to the variability of neuropathology and proteinopathy endophenotypes within AD brains^8,12,13^ or across disease progression.^14,15^ Results of these studies have deepened and refined our understanding of the gene expression mechanisms that underpin distinct features of AD. A recent study investigated transcriptomic changes in AD brain tissue using laser capture microdissection around Aβ plaques and NFTs finding gene expression changes related to neuroinflammation and neuronal dysfunction pathways mainly around Aβ plaques rather than NFTs.^16^ In contrast, another study using multi-omic integration approaches with gene expression and histone acetylation data found epigenetic and corresponding transcriptomic changes specifically in relation to tau but not Aβ burden.^17^ Understanding gene expression dysregulation associated with proteostasis in the AD brain is important because different proteins core to the neuropathological hallmarks of AD, namely Aβ, tau and APOE, and their distinct brain biochemical states may have distinct functions in the pathophysiology of AD.^18-20^

Indeed, analyses of these proteins in brain as well as fluid and imaging AD biomarkers have revealed complex and dynamic changes in their biochemical states, which correlate to an extent with the clinical progression of AD.^18,21^ In AD brains, insoluble biochemical states of Aβ40, Aβ42, and APOE are components of senile plaques, while soluble levels of total tau rise, become hyperphosphorylated (pTau), and then insolubilized into NFTs with AD progression. Insoluble NFTs composed of pTau correlate with cognitive decline.^22,23^ Furthermore, tau oligomers can bind plasma membranes^24-29^ and increase tau fibrillization through this membrane interaction.^30-33^ The levels of and ratio between Aβ40 and Aβ42 change in brains of patients with AD and animal models.^29,34-38^ Additionally, Aβ oligomers have the ability to bind and cause lipid membrane disruptions.^39-42^ In carriers of *APOE*ɛ4, the strongest genetic risk factor for AD,^43^ there is a shift of APOE from membrane-bound to more soluble and insoluble biochemical states.^44,45^

Using neuropathology and brain biochemical levels of core AD proteins as endophenotypes, we previously conducted a genome wide association study (GWAS)^20^ and an epigenome-wide association study of DNA methylation (DNAm)^46^ in AD brains. We identified genetic variants and regional DNAm changes that are commonly associated across multiple AD endophenotypes, as well as those unique to specific measures. These findings begin to uncover molecular pathways linked to these brain AD endophenotypes. Nonetheless, transcriptome-wide gene expression changes associated with specific brain biochemical states of core AD proteins remain to be uncovered. Furthermore, an integrative analysis of genetic variants, brain transcriptome and epigenome can yield potential regulatory mechanisms that may underlie the variability in the complex biochemical states of these proteins that are critical to AD pathophysiology.

To identify biological pathways and molecular mechanisms that may influence these core AD brain endophenotypes, we conducted a brain transcriptome-wide association study (TWAS) of AD neuropathologic measures including Braak stage, Thal phase, cerebral amyloid angiopathy (CAA) score and brain tissue levels of five AD-related proteins (APOE, Aβ40, Aβ42, tau and pTau) in a cohort of 477 AD donors. We integrated our findings with genetic variant and brain DNAm data from the same donors and annotated for biological pathway and cell-type enrichment. We identified common as well as distinct gene expression and network associations with multiple core AD endophenotypes revealing transcriptome dysregulation especially relating to brain tau levels that likely reflect molecular pathways underlying AD pathophysiology. Based on these findings, we propose a novel hypothetical model of *dynamic brain gene expression changes* that track with progressive AD proteostasis. These expression changes hold potential to serve as dynamic, precision biomarkers of brain AD progression.

## Materials and methods

### Brain donors and samples

Postmortem superior temporal cortex (TCX) tissue samples were collected by the Mayo Clinic Brain Bank (Jacksonville, FL, USA) and are a part of the Mayo Clinic AD-CAA (MC-CAA) study. Cohort information has been described previously^3,20^ and is detailed on the AD-knowledge portal (https://adknowledgeportal.synapse.org, see data sharing section). All samples had a confirmed AD neuropathological diagnosis defined as a Braak stage ≥ four and Thal phase ≥ two, were scored for CAA pathology, had an age at death greater than 55 years, and due to availability, were all non-Hispanic white (Supplementary Table 1). Individuals with co-pathologies were not excluded to allow for inclusion of the full spectrum of neuropathological disease common to AD. This study was approved by the appropriate Mayo Clinic Institutional Review Board.

### Endophenotypes

#### Neuropathology

Braak stage, Thal phase and CAA scores were measured by one neuropathologist (D.W.D.) in the Mayo Clinic Brain Bank using previously established protocols.^19,23,47-49^ Intermediate Braak stages were grouped with the next lowest stage as follows, stage 3.5 is 3, 4.5 is 4 and 5.5 is 5 as detailed previously^3,19^ to allow for comparisons with other datasets that do not have intermediate Braak stages (Supplementary Table 1, Supplementary Fig. 1).

#### Biochemical measures

A collection of biochemical measures from a subset of superior temporal cortex brain samples included in the MC-CAA study has been described previously.^19,20^ These measures include five AD-related proteins [APOE, Aβ40, Aβ42, tau and phospho-tau (Thr231)] from three tissue fractions (soluble, membrane-bound and insoluble). Briefly, supernatant fractions were collected after three sequential buffer treatments of tissue homogenate and resulting pellets: first with tris-buffered saline buffer (TBS), second with detergent (TBS/1% Triton X) buffer (TX), and lastly with formic acid (FA) buffer. Protein quantification was performed via ELISA for each fraction and normalized against total protein quantities. Biochemical measures were transformed by either the natural log or square root to approximate a normal distribution, including the Aβ40/42 ratio. Measures were then standardized with a mean of zero and standard deviation of one using the z-score formula to allow for comparisons of effect sizes between biochemical measure analyses (Supplementary Fig. 1). Of note, in this study, we explore APOE protein levels, *APOE* gene expression levels and *APOE*ε2/3/4 genotype data.

### Gene expression

Bulk RNAseq data were generated for 477 TCX samples as part of the MC-CAA study. Data generation and quality control are described in detail elsewhere.^46^ Briefly, total RNA was extracted using Trizol® reagent and cleaned using Qiagen RNeasy columns with DNase treatment. Randomized samples with a RNA integrity number (RIN) > 5.5 (Supplementary Fig. 1) were sent to the Mayo Clinic Medical Genome Facility Gene Expression and Sequencing Cores for library preparation and sequencing on the Illumina HiSeq 4000 (Illumina). For quality control, the Mayo Clinic MAP-Rseq pipeline v.3.0.2^50^ implementing STARv2.5.2b^51^ was used for read alignment to hg38 and featureCounts from the Subread package v1.5.1 for counting.^52^ Raw read counts were log2-transformed and normalized using conditional quantile normalization (CQN) via the Bioconductor package; accounting for sequencing depth, gene length, and GC content.^53^ FastQC and RSeQC^54^ were used for QC of raw and mapped reads. Samples with <50 million reads mapped to genes, <50% of reads mapped to genes, that were outliers (1.5 inter-quartile range) based on distribution of % junction reads, that failed sex check, that were outliers (>4 SDs from the mean) on principle components (PCs) 1 and 2 in a PCA (*prcomp*, CPM, R statistical software), or that failed previous genetic based QC steps^3^ were excluded. There were 456 TCX RNAseq samples that passed QC.

### DNA methylation

DNA methylation measures were generated and quality controlled previously and are described in detail elsewhere.^46^ Briefly, DNA isolated from the TCX was processed via Reduced Representation Bisulfite Sequencing (RRBS) methods by the Mayo Clinic Genome Analysis Core (GAC) in Rochester, MN following manufacturer’s recommendations for the NuGen Ovation RRBS Methyl-Seq System 1-16 (Tecan Genomics) and sequenced on the HiSeq 4000 (Illumina). Raw sequencing reads were analysed using the Streamlined Analysis and Annotation Pipeline for Reduced Representational Bisulfite Sequencing (SAAP-RRBS)^55^ pipeline. Additional quality control was performed on the sample and CpG levels. Regional levels of CpGm (rCpGm) were then calculated using the ChromHMM core 15-state chromatin model developed by the Roadmap Epigenomics Consortium^56^ for the adult brain Inferior Temporal Lobe (E072). All CpGm sites that passed QC were grouped into regions based on the 15-state chromatin model annotations and averaged to determine rCpGm levels. rCpGm start and stop sites were defined as the first and last CpGm present in each rCpGm group. Data reduction steps were then performed to remove non-variable rCpGms that are not statistically informative and that would unnecessarily contribute to the multiple testing correction burden.

### Genotype data

Genome-wide genotypes (GWGs) were generated in previous studies and are described in detail elsewhere.^3,20^

### AMP-AD dataset

The TCX Mayo RNAseq study^57^ was obtained from the AD-knowledge portal (https://adknowledgeportal.synapse.org). The RNA-seq data previously underwent consensus reprocessing by Accelerating Medicines Partnership-AD (AMP-AD, RNAseq Harmonization Study)^4^ and additional QC of this data, including harmonization of diagnosis based on neuropathological measures, is described in detail elsewhere.^3^

### Statistical analysis

#### TWAS, MC-CAA

There were three samples with a Thal phase measure of 2 that passed RNAseq QC. Given the low number of this measure in a single group and the possibility that small variations in this group may considerably and unreliably impact our statistical estimates, we set these Thal phase 2 measures to *NA* in analyses that included Thal phase as a variable. We tested the association of normalized gene expression data with the endophenotype of interest through multi-variable regression in R. We defined genes to be expressed based on the distribution of CQN values and only analysed those genes with a median CQN value greater than or equal to 1. We ran the following linear regression model: cqn(gene expression) ∼ endophenoytpe + Age at Death + sex + RIN + (1|flowcell) using the *lmer* function in the *lmerTest* package with ordinal variables (Braak stage and Thal phase) coded as an ordered factor.^58^ Bonferroni *P*-value threshold was calculated by number of genes tested: (0.05/18383) = 2.719904E-06. FDR *P*-values were calculated using *p.adjust()* and the Benjamini-Hochberg FDR procedure. Sensitivity analyses with AD neuropathology measures included three additional binary (presence/absence) covariates consisting of Braak stage 5, Braak stage 6, and the combination of Thal phase 4 and 5. Additional sensitivity analyses were performed using the *lmer* function adding neuronal cell type proportion estimates in the model and removing the RIN covariate from the model, as well as Spearman’s rank correlation tests in R with the *cor.test()* function of the *stats* package.

#### TWAS, AMP-AD

We investigated the association between CQN normalized gene expression values with AD diagnosis (*N* = 80 ADs, *N* = 68 Control) adjusting for age at death, sex, RIN, flowcell and tissue source (Banner or Mayo Clinic) using linear regression implemented in R statistical software. As described previously, we defined genes to be expressed based on the distribution of CQN values and only analysed those genes with a median CQN value greater than or equal to 0.5.^3^

### Pathway enrichment

Pathway enrichment was tested using the *anRichment* v1.26 package in R with the Gene Ontology Biological Processes database from the *org.Hs.eg.db* v3.18.0 package. *P*-values were computed via hypergeometric tests and FDR *P*-values using the Benjamini-Hochberg FDR procedure. The background gene set consisted of all autosomal genes that are reliably expressed above background in each tissue type.

### Weighted gene co-expression analysis

For analysis of transcriptome co-expression networks, the weighted gene co-expression network analysis (WGCNA) package^59^ was used to construct gene modules for analysis using normalized gene expression residuals of expressed genes after adjusting for technical and biological covariates including Age at Death, sex, RIN, and (1|flowcell). A soft power threshold was selected (*pickSoftThreshold*) based on an R-squared value of 0.9 to ensure a scale-free topology of the resulting network. Following this, a signed network was constructed and the resulting topological overlap matrix (TOM) clustered to generate initial modules which were then refined through cutting of the dendrogram (*blockwiseModules*). Module eigengenes were computed and then correlated with disease and neuropathological phenotypes using Spearman’s rank correlation tests adjusting for the number of tests using Bonferroni correction. We annotated the modules by calculating enrichment of biological process (BP) gene ontology (GO) terms as described in the ‘Pathway enrichment’ section as well as cell type marker gene enrichment described in the ‘Cell type marker gene enrichment’ section using the top 100 BRETIGEA cell-type marker genes.

### eQTL analysis

*Cis-*expression quantitative trait locus (eQTL) analysis between imputed genetic variant dosages lifted over to hg38 and gene expression measure residuals was performed in R using the *MatrixEQTL*^60^ package adjusting for biological covariates including age at death, sex and the first three genetic principle components. Gene expression residuals were calculated by regressing out technical covariates including RIN as a fixed effect and flowcell as a random effect using the *lmer* function in R. *Cis*-eQTLs were defined as SNPs and genes ±500 kb apart.

### EWAS, *cis*-methylation QTL and eQTM associations

The EWAS, *cis*-methylation QTL (mQTL) and eQTM analyses are described in detail elsewhere.^46^ False discovery rate (FDR) corrected *P*-values were calculated with the R *p.adjust* package using the Benjamini–Hochberg method.

### Cell-type proportion estimates

Cell-type proportions from the bulk tissue RNAseq data were estimated using the Digital Sorting Algorithm (DSA)^61^ via the *DSA* R package (v1.0) and using the top 100 genes from BRETIGEA^62^ as the cell type–specific marker genes. Oligodendrocyte precursor cell (OPC) types were not investigated, as we previously did not observe robust correlations among the defined OPC marker genes, as described in Wang *et al.*^5^

### Cell-type marker gene enrichment

One-sided hypergeometric tests using the *phyper(lower.tail = FALSE)* function from the R *stats* package were used to test for enrichment of cell-type marker genes defined by BRETIGEA^62^ in our dataset. We tested the top 100 and/or top 1000 defined cell type marker genes as specified using the total number of autosomal genes reliably expressed above background tested in our TWAS analyses as the total universe number.

## Results

### Brain tau levels associate with the brain transcriptome

Using a well-characterized dataset of neuropathologically confirmed AD donors from the Mayo Clinic Brain Bank, we investigated the association of TCX gene expression levels with neuropathological and biochemical measures of core AD endophenotypes. After stringent QC of the 57 353 genes profiled in the TCX, our final dataset consisted of 456 TCX samples with 18 383 autosomal genes reliably expressed above background (Supplementary Table 1). Notably, gene expression and biochemical measures were obtained from proximal brain tissue samples.

**Figure 1:**
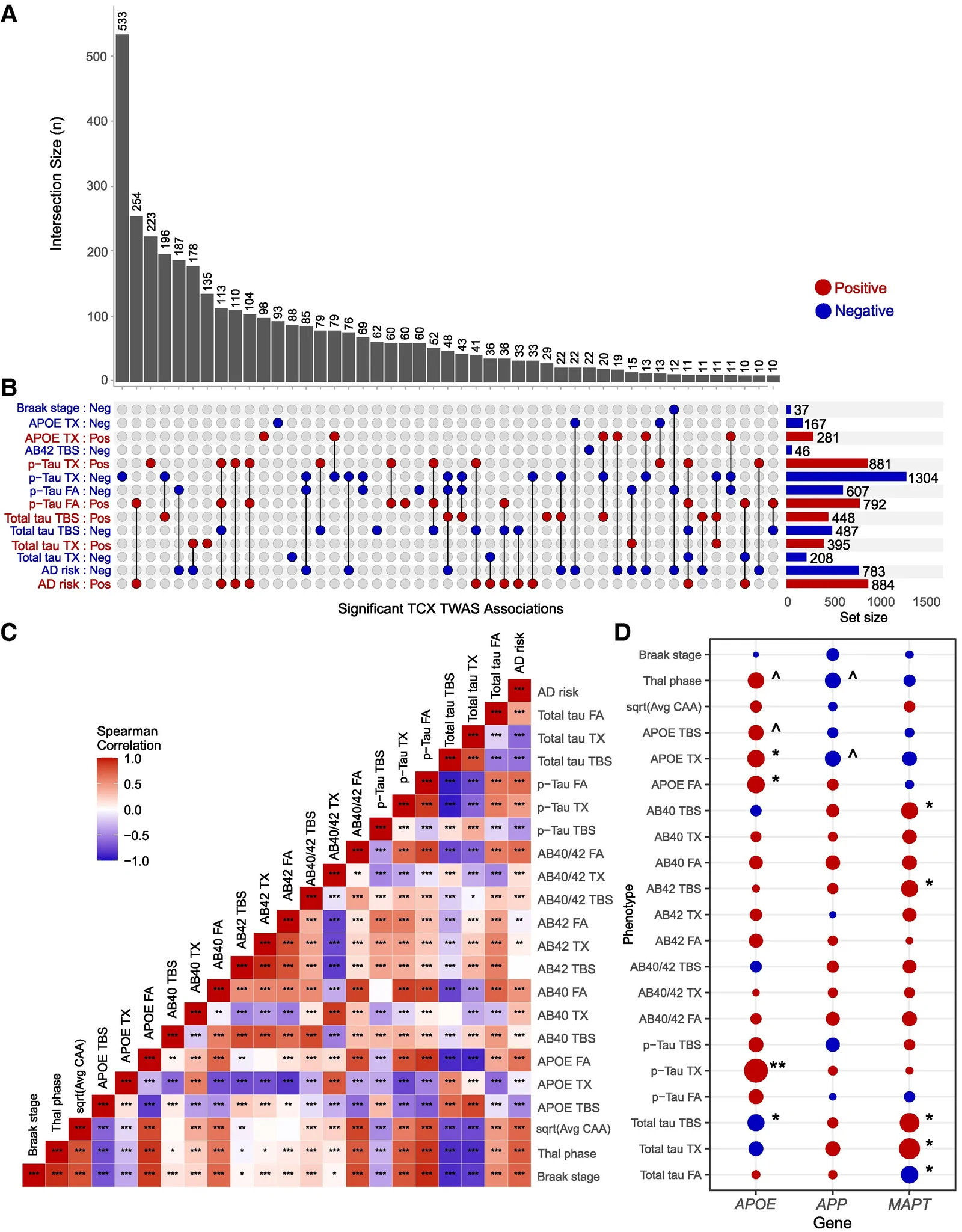
**Summary of significant temporal cortex TWAS associations:** Upset plot showing Bonferroni significant (Bonferroni *pval ≤* 0.05) gene expression associations tested via linear regression across each endophenotype with (**A**) showing the total intersection set numbers and (**B**) showing the intersections for each phenotype as well as nominal AD risk associations (*pval* ≤ 0.05) from the AMP-AD Mayo RNAseq TCX dataset. Sets less than nine are not shown. An extended figure with all sets can be found in Supplementary Fig. 2. (**C**) Spearman’s correlations between the regression beta values for each of the 3835 implicated genes across all endophenotypes and AD risk. (**D**) TWAS linear regression results of temporal cortex *APOE*, *APP* and *MAPT* gene expression with each temporal cortex endophenotype. Biochemical endophenotypes were transformed by either natural log or square root and standardized, see Methods for more details. *** = *pval* ≤ 1E-05; ** = 1E-05 ≤ *pval* ≤ 0.001; * = 0.001 *≤*  *pval* ≤ 0.05; ^ = 0.05 ≤ *pval* ≤ 0.1. FA = insoluble formic acid fraction; GE = gene expression; *N* = number; pTau = phospho-tau; TBS = soluble tris buffered saline fraction; tTau = total tau; TWAS = Transcriptome wide association study; TX = membrane associated Triton-X fraction.

We tested the association of gene expression levels with the neuropathological variables of Braak stage, Thal phase and CAA scores as well as with 18 TCX biochemical measures of five proteins,^19^ namely APOE, Aβ40, Aβ42, total tau and phospho-tau (pTau and Thr231) extracted from the soluble (TBS), membrane-bound (TX) and insoluble (FA) tissue fractions, as well as the ratio of Aβ40/42 (Supplementary Fig. 1). We identified 5740 Bonferroni significant gene expression-endophenotype associations (*P*-value ≤ 2.719E-06), with 3835 unique genes in our TWAS (Fig. 1, Table 1, Supplementary Fig. 2, Supplementary Fig. 3). There were 5655 significant associations with 12 biochemical endophenotypes, 68 with Braak stage and 17 with Thal phase. Brain levels of pTau had the highest number of significant associations (*N* = 3,595, 62.6%) and total tau the second highest (*N* = 1,545, 26.9%). Aside from the tau-related associations, we also found 460 gene expression associations with APOE, 54 with Aβ42 and 1 with Aβ40_TBS_.

**Table 1:** Top TCX TWAS associations: Summary of Bonferroni-significant TCX TWAS associations

Endophenotype	Total *N*	Beta Value (*N*)		Top 5 significant genes		Top GO BP Gene set enrichment		Cell type marker gene enrichment	
		Pos	Neg	Pos	Neg	Pos	Neg	Pos	Neg
Braak stage	68	31	37	*CIC, PIRT, MED13L*	*RP11-321E2.3, SLC2A11*	NS	NS	NS	NS
Thal phase	17	9	8	*CIC, GFAP*	*ZNRF1, POLR3K, C12orf73*	NS	NS	NS	NS
Total tau TBS (sqrt and std)	935	448	487	*ZC3H8, BCRP3*	*OLFML3, ECM2, MFAP4*	Nucleic acid metabolic process	Immune system process, cell adhesion	NS	Mic, End
Total tau TX (sqrt and std)	603	395	208	*RP11-309L24.4, RP11-465B22.3*	*COPZ2, APH1A, ZNFX1*	synaptic signalling, regulation of transport	NS	Neu	End
Total tau FA (sqrt and std)	7	4	3	*FTL, PIK3R2, FTH1*	*RP11-192H23.5, DIABLO*	NA	NA	NA	NA
p-Tau TBS (sqrt and std)	11	6	5	*RP4-738P15.6*	*UBN1, SPECC1L-ADORA2A, SH3D21, RC3H2*	NS	NS	NS	NS
p-Tau TX (ln and std)	2185	881	1304	*RP11-283G6.5*	*MC1R, HINFP, SIRT6, FSD2*	cell adhesion, cell migration, response to wounding, immune response, cell communication	Nucleic acid metabolism, RNA processing, gene expression, DNA repair and metabolic process, response to DNA damage	Ast, Mic	NS
p-Tau FA (ln and std)	1399	792	607	*CIC, ECM2, ITPKB*	*RPH3A, KDM2B*	wound healing, cell adhesion, cell motility, cell communication and signalling, vasculature development	ATP biosynthetic process, oxidative phosphorylation, mitochondrial transport chain complex assembly	Ast, End	NS
AB42 TBS (ln and std)	49	3	46	NA	*RPS13P2, EPM2A, RWDD1, PAQR6, RP11-380M21.2*	NA	NS	NA	Oli
AB42 TX (ln and std)	1	0	1		*RPS13P2*	NA	NA	NA	NA
AB42 FA (ln and std)	4	3	1	*NCAN, HIGD1A, RP4-683L5.1*	*ZMYND10*	NA	NA	NA	NA
AB40 TBS (ln and std)	1	0	1		*THADA*	NA	NA	NA	NA
APOE TX (sqrt and std)	448	281	167	*ZNF431, ZBTB25*	*RP11-82L18.4, SHC3, SHOC2*	Cellular response to DNA damage stimulus	Regulation of biological quality	NS	NS
APOE FA (ln and std)	12	4	8	*APLNR, SGCB, DDR1*	*CTD-2369P2.10, CTBP1*	NA	NS	NA	NS

Number of significant associations by effect direction and effect magnitude and the top 5 most significant genes by effect direction. Significance determined by Bonferroni *P*-value ≤ 0.05 from primary TWAS linear regression models. Further characterization of Bonferroni-significant results was performed through gene set and cell-type marker gene enrichment analyses conducted only when gene sets included 5 or more genes. GO BP = Gene Ontology Biological Processes; *N* = number; NS = not significant; NA = not applicable; ln = natural log; sqrt = square root; std = standardized; TCX = temporal cortex; TWAS = transcriptome-wide association study; pos = positive beta (>0); neg = negative beta (<0); Mic = microglia; End = endothelia; Ast = astrocyte; Neu = neuronal; Oli = oligodendrocyte.

We first investigated whether the significant TWAS gene transcripts were associated with overall levels of an AD-related protein or with a specific tissue fraction, i.e. soluble (TBS) versus membrane-bound (TX) versus insoluble (FA). Interestingly, none of the significant genes were associated with all three fractions of a single protein. Most overlaps occurred with TWAS results significant for specific brain tau fractions, particularly between pTau_TX_ and total tau_TBS_ that were in opposite directions (Fig. 1, Supplementary Figs 2 and 4). There were 29 brain transcripts commonly associated with total tau_TBS_, total tau_TX_, pTau_TX_ and pTau_FA_, including *ABCB6*, *DDAH2*, *TRIM27, VCAM1, SLC12A4* and *PUS1*, where associations with pTau fractions were in opposite directions to those of total tau (Supplementary Figs 2 and 4). We also examined genes common to a specific fraction type across the tau proteins and found only 2 genes in the insoluble fraction (total tau_FA_, pTau_FA_), namely *DIABLO* and *MSMP*. No genes were commonly significant across the APOE fractions, and only one gene, *RPS13P2*, was significant across two fractions of Aβ (Aβ42_TBS_ and Aβ42_TX_) (Supplementary Fig. 4). These results suggest opposite relationships between pTau and total tau fractions on common sets of genes, but unique gene expression association patterns with APOE and Aβ protein fractions.

We also tested the robustness of the biochemical endophenotype associations (*N* = 5655) to influences of neuropathology levels, neuronal cell-type proportion estimates, RIN or presence of potential outliers by performing sensitivity tests. To do this, we additionally adjusted for Braak stage and Thal phase as well as estimated neuronal cell-type proportions, excluded RIN as a covariate, and finally, performed a non-parametric Spearman’s rank test. After adjusting for Braak stage and Thal phase, all biochemical measure associations remained at least nominally significant (*P*-value ≤ 0.05) with consistent effect estimates and 54% remained Bonferroni significant (Supplementary Fig. 5). The greatest drop in significance, following neuropathology level adjustments, occurred with insoluble pTau (pTau_FA_) TWAS, although there was still a considerable number of Bonferroni-significant associations even after adjusting (*N* = 439; 31.4%). Braak stage is a measure of NFTs in the brain composed of insoluble pTau, which likely explains the drop in significance for this TWAS after neuropathology adjustments. However, although the AD biochemical and neuropathological endophenotypes are correlated, most TWAS results are not driven by neuropathology alone. We next adjusted for neuronal proportions using deconvolution methods to estimate neuronal cell-type proportions from bulk tissue gene expression measures. We found that all but 50 total tau_TX_ associations remained at least nominally significant and 62% remained Bonferroni significant, all with consistent directions of effect. We see the greatest reductions in associations with Aβ42_FA_, Aβ42_TBS_, APOE_FA_, APOE_TX_, total tau_TX_ and pTau_FA_; however, we see robust preservation in test statistics for associations with Aβ40_TBS_, Aβ42_TX_, total tau_FA_, pTau_TBS_ and pTau_TX_. Furthermore, because previous work^63^ has suggested correcting for RIN may overcorrect if phenotypes are related to disease severity, we removed RIN from our model finding 89% remained Bonferroni significant (*P*-value ≤ 0.05). In our Spearman’s rank test, all but three associations were at least nominally significant (*P*-value ≤ 0.05), and all had consistent effect directions. These results suggest that our associations are not driven by neuronal proportion changes in AD nor phenotype or gene expression outliers.

### Brain transcriptomic changes associated with tau are reflective of AD-related changes

We next hypothesized that if the TCX brain biochemical measure associations are reflective of AD-related changes in the brain transcriptome, then there will also be biologically congruent differential expression of these genes between AD and control brains in the same brain region. To test this, we compared our brain biochemistry TWAS associations to AD diagnosis associations in an independent dataset from the AMP-AD Mayo TCX RNAseq cohort.^57^ Biological congruency was based on previously defined relationships between AD biochemical and neuropathology measures.^19,20,46^ For example, pTau_TX_ positively associates with AD neuropathologic measures of Braak stage (tau) and Thal phase (Aβ). Therefore, brain transcripts that positively associate with both pTau_TX_ in our TWAS and with AD diagnosis in the independent AMP-AD Mayo TCX RNAseq cohort^57^ would be deemed to have biologically congruent brain endophenotype and AD associations. We found that 1667 unique genes with 2627 significant TWAS associations were also differentially expressed (*P*-value ≤ 0.05) in AD versus control brains, 2435 of which were biologically congruent. This suggests that nearly half of the TCX brain biochemistry associations (42.4% = 2435/5740) may be reflective of AD-related changes (Fig. 1A and B, Supplementary Fig. 2).

To investigate correlation patterns between each significant TWAS and AD differential gene expression associations, we analysed beta values of association for the 3835 unique genes from the 5740 significant TWAS. When beta values for these 3835 genes are correlated across all their endophenotype associations, significant patterns emerge (Fig. 1C). There are significant positive correlations between beta values of gene expression associations across insoluble fractions (FA) of most biochemical measures and both AD neuropathology and diagnosis. This suggests that there may be underlying disease mechanisms relating to gene expression changes occurring with these insoluble aggregated proteins which increase in AD (Fig. 1C). In contrast, there are strong negative associations between the TWAS beta values for soluble (APOE_TBS_, pTau_TBS_ and total tau_TBS_) and membrane-bound (total tau_TX_) brain biochemical measures with both AD neuropathology and diagnosis. This suggests that mechanisms that promote soluble or membrane-bound fractions of these biochemical measures may be protective against AD. Alternatively, these brain biochemical fractions may be reduced with AD.

Next, because the APOE, Aβ and tau proteins are encoded by the *APOE, APP* and *MAPT* genes, respectively, we investigated the association of these proteins with expression levels of their encoding genes (Fig. 1D). None of these three genes had Bonferroni-significant associations with any of the fractions of proteins that they encode; however, there were positive nominal associations (*P*-value ≤ 0.05). Brain *APOE* levels are nominally associated with increased APOE_TX_ and APOE_FA_. On the other hand, *MAPT* expression is nominally associated with increased levels of total tau_TBS_ and total tau_TX_ and decreased with total tau_FA_, but not with any fractions of pTau. Interestingly, *APP* levels were not significantly associated with Aβ fractions but were negatively trending with Thal phase (*P*-value = 0.07), a measure of amyloid pathology. These results suggest that there are likely other mechanisms that impact tissue fraction-specific levels of these proteins beyond that of their coding genes’ expression.

### Pathway and gene co-expression network analysis implicates brain tau levels in dysregulation of gene networks

Subsequently, we sought to identify biological pathways and cell types enriched in each TWAS, as this can provide insights into the molecular and cellular changes occurring with these brain neuropathology and biochemical measures. Gene Ontology (GO) pathway and cell type marker gene enrichment analyses were performed for significant TWAS genes separately for each endophenotype split by the direction of effect when there were at least five significant genes in a group (Table 1, Supplementary Table 2). Genes upregulated with total tau_TBS_ and down regulated with pTau_TX_ were both enriched in pathways related to nucleic acid metabolic processes. In contrast, downregulated total tau_TBS_ and upregulated pTau_TX_ genes showed enrichment in immune-related and cell adhesion pathways as well as enrichment for microglia cell-type marker genes. pTau_FA_ showed upregulation of pathways related to cell adhesion, wound healing and vascular development along with enrichment of endothelial and astrocyte cell-type marker genes, while downregulated pTau_FA_ pathways are related to mitochondrial function. Finally, APOE_TX_ showed upregulation in cell response to DNA damage and downregulation of cellular localization–related pathways.

**Figure 2:**
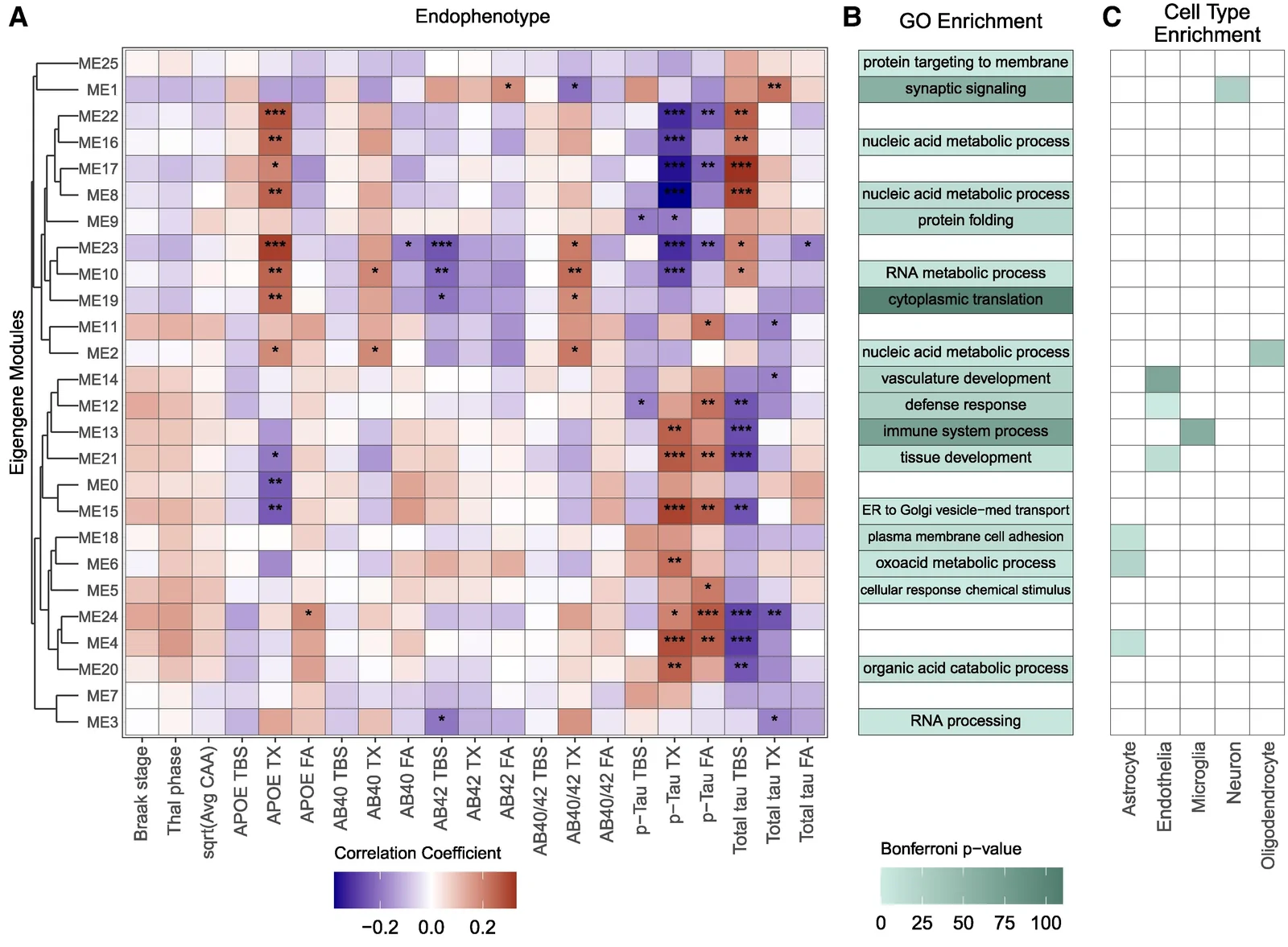
**Broad dysregulation of temporal cortex gene co-expression networks relate to brain tau levels:** (**A**) Spearman’s correlations between eigengene modules (ME) from the TCX weighted gene co-expression network analysis and each endophenotype assessed. Blue indicates negative correlations and red positive. Asterisks denote Bonferroni *P*-value after correction for 234 tests (nine protein sets * 26 MEs): *P-value < 0.05, **P-value < 0.001, ***P-value < 1E-05. (**B**) ME genes were assessed for enrichment of Gene Ontology (GO) Biological process terms using a hypergeometric test. Top GO terms were manually curated for the plot; a full list of enriched GO terms can be found in Supplemental data. Colour denotes the strength of the Bonferroni *P*-value. (**C**) Enrichment of top 100 cell type marker genes defined by BRETIGEA was assessed for each ME via hypergeometric tests. Colour denotes the strength of the Bonferroni *P*-value. FA = insoluble formic acid fraction; GO = Gene Ontology; ln = natural log; pTau = phospho-tau; sqrt = square root; TBS = soluble tris-buffered saline fraction; tTau = total tau; TX = membrane-associated Triton-X fraction.

We also investigated co-expression of gene networks associated with each AD endophenotype, constructing co-expression networks with WGCNA,^59^ finding a total of 26 TCX co-expression modules (MEs). Using Spearman’s correlation, we tested the association between MEs and each AD endophenotype, finding 70 Bonferroni-significant associations (Fig. 2). Remarkably, we found that our WGCNA results largely recapitulated our TWAS pathway enrichment analyses, including associations largely with total tau and pTau often with opposite directions of effect enriched in GO terms relating to nucleic acid and RNA metabolic processes, immune and defence responses, tissue development and organic acid catabolic processes (Fig. 2). Interestingly, we observed that the ME dendrogram splits into two main groups, one that generally associates positively with the neuropathology measures (Fig. 2A, ME11-ME3 top to bottom) and the other negatively (Fig. 2A, ME25-ME19 top to bottom). These two module groups have patterns of associations with the other measures that are aligned with previously described correlations between these brain AD endophenotypes.^19,20,46^ The MEs associate most strongly and broadly with levels of APOE_TX_, pTau_TX_, pTau_FA_ and total tau_TBS_ (Fig. 2). MEs that associate with lower neuropathology also have negative associations with pTau_TX_ and pTau_FA_ but associate positively with APOE_TX_ and total tau_TBS_. In opposite directions, these brain endophenotype associations are observed with MEs that have positive associations with AD neuropathology.

Together, these results suggest that there is a strong and inverse relationship between levels of membrane-bound APOE_TX_ and soluble total tau versus membrane-bound and insoluble pTau (pTau_TX_ and pTau_FA_), which associate with dysregulation of gene expression networks in the TCX of the AD brain. Moreover, each AD-related brain biochemical measure has a composition of shared and unique gene pathways and enriched cell types underlying their variability in the brain.

### Brain cell-type proportions associate with biochemical endophenotypes

**Figure 3:**
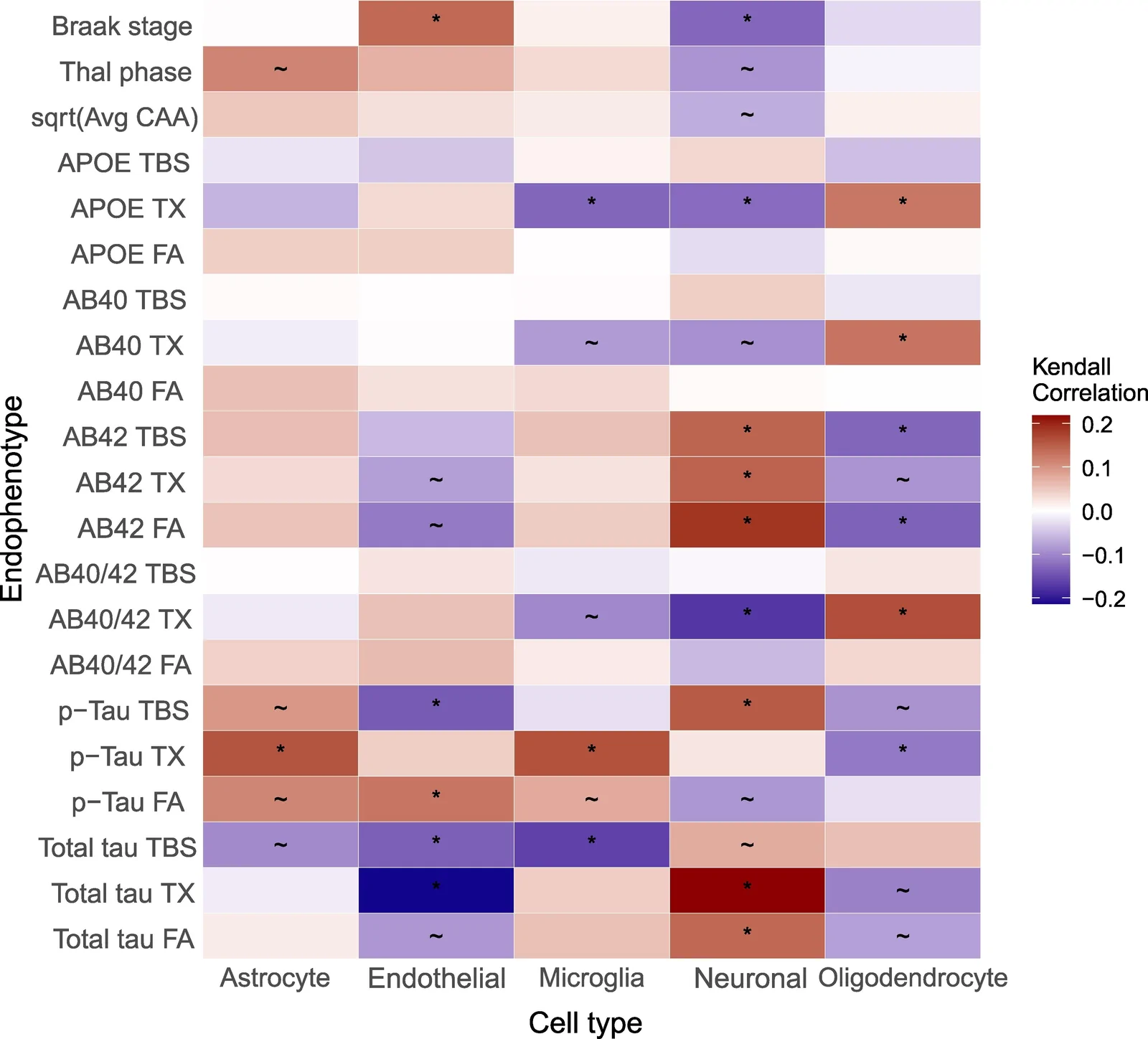
**Brain cell-type proportions associate with biochemical endophenotypes:** Each square represents the Kendall correlation between the estimated cell-type proportion (x-axis) and each endophenotype (y-axis). Blue indicates negative correlations and red positive. (*) indicates a Bonferroni *P*-value ≤ 0.05, (∼) indicates a nominal *P*-value ≤ 0.05. FA = insoluble formic acid fraction; ln = natural log; pTau = phospho-tau; sqrt = square root; TBS = soluble tris buffered saline fraction; TCX = temporal cortex; tTau = total tau; TX = membrane associated Triton-X fraction.

Subsequently, because we found enrichment of specific cell types with individual endophenotypes, we asked if estimated cell-type proportions correlate with the biochemical endophenotypes to investigate potential endophenotype specific cell-type vulnerabilities. Proportions of neurons, astrocytes, microglia, oligodendrocytes and endothelial cells were estimated with DSA deconvolution methods^61^ using the top 100 BRETIGEA cell-type marker genes^62^ and correlated with each endophenotype via Kendall rank tests (Fig. 3, Supplementary Table 3, Supplementary Fig. 6). Most Bonferroni significant cell proportion associations were with the tau measures including positive associations of pTau_TX_ with astrocytes and microglia, pTau_FA_ with endothelial, as well as pTau_TBS_, total tau_TX_ and total tau_FA_ with neuronal cell types. We found significant negative associations between total tau_TBS_ with microglia and endothelial, as well as total tau_TX_ and pTau_TBS_ with endothelial cell types. Additionally, we found nominal associations of neuronal proportions with neuropathology levels which align with known reductions in neuronal cell populations with higher neuropathology levels in AD. Interestingly, neuronal proportions showed positive associations across the fractions of Aβ42 despite insoluble Aβ42 being a major component of Aβ plaques. This potentially suggests that TCX neuronal proportions are impacted by factors that are integral to the formation of Aβ plaques beyond just an increase in levels of insoluble Aβ. Comparisons between Aβ and tau species show that Aβ may impact (or be impacted by) neuronal and oligodendrocyte levels, while tau may influence (or be influenced by) astrocyte, endothelial, microglial and neuronal proportions. Importantly, however, these associations are unable to provide casual directionality, may be secondary effects of neurodegenerative processes, and it remains unclear which cell types may be of greatest importance as a change in the relative proportion of one implies changes in others. Nonetheless, these results suggest that changes in TCX cell proportions are not driven by levels of a single protein investigated here but are likely influenced by a complex interplay between levels of these proteins and factors beyond these measures.

### Gene expression measures associate with proximal DNA methylation in an endophenotype specific manner

**Figure 4:**
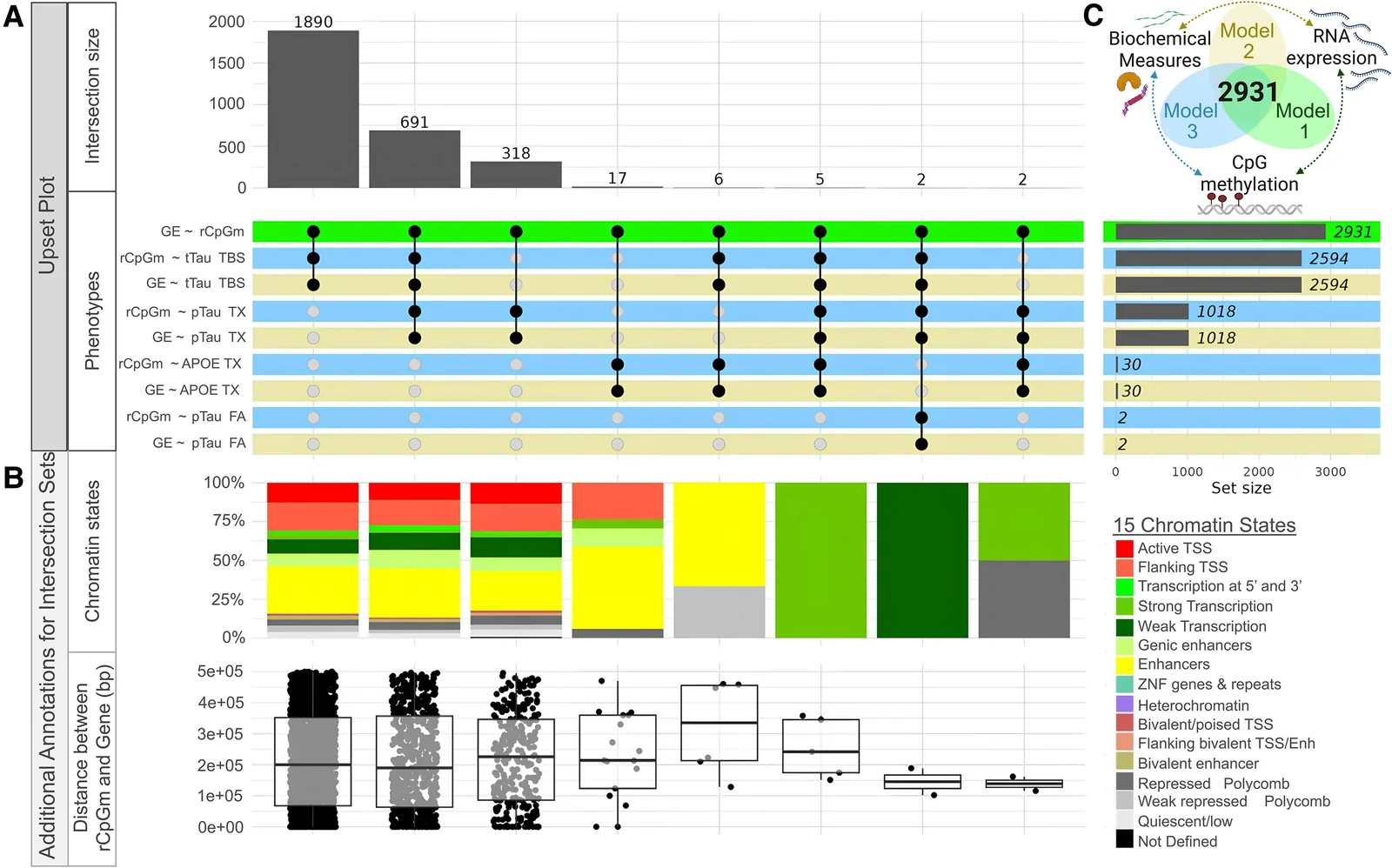
**Concordant associations across multiple rCpGm, gene expression, and endophenotype association models:** (**A**) Upset plot showing rCpGm-gene expression (GE)-phenotype triplets that were FDR significant (FDR *pval* ≤ 0.05) across three linear regression association models tested in the temporal cortex, including Model 1: GE ∼ rCpGm, Model 2: GE ∼ pheno and Model 3: rCpGm∼pheno. rCpGm-GE-pheno triplets, which showed discordant association effect direction patterns across the three models, were removed (*N* = 20 triplets with tTau TBS). See Article text. (**B**) Bar and box plots show additional annotations for each intersection set. Chromatin states depict the Epigenetic Roadmap 15-chromatin state model annotation breakdown for each rCpGm of the intersection set. A box and whisker plot depicts the distance in base pairs (bp) between the rCpGm and gene pair at their closest point. (**C**) Graphical schematic illustrating the 3 different models tested for (**A**) and (**B**) to identify the 2931 unique *cis-*eQTM pairs (Model 1) with concordant associations in Models 2 and 3. (**C**) Created in BioRender.com. Oatman, S. (2026) https://BioRender.com/gwbkfog. FA = insoluble formic acid fraction; FDR = false discovery rate; GE = gene expression; *N* = number; pheno = endophenotype used in association tests, pTau = phospho-tau; rCpGm = regional CpG methylation; TBS = soluble tris buffered saline fraction; tTau = total tau; TX = membrane associated Triton-X fraction.

To uncover potential mechanisms of expression dysregulation related to the AD endophenotypes, we analysed DNA methylation (DNAm) measures available from the same brain regions of the same donors in our TWAS. We hypothesized that these brain expression dysregulations may be driven by DNAm changes within proximity to these genes. To investigate the relationship between gene expression dysregulation and levels of proximal regional DNAm (rCpGm), we utilized TCX rCpGm levels obtained from these same donors.^46^ We examined 1 612 499 TCX *cis*-rCpGm-gene pairs ±500 kb apart using expression quantitative trait methylation (eQTM) analysis. From this eQTM analysis, we identified 12 973 TCX FDR significant *cis-*eQTM associations (FDR *P*-value ≤ 0.05). The most significant associations were often in proximity with negative effect directions, but the eQTMs spanned the entire ±500 kb window with both effect directions (Supplementary Fig. 7). The majority of the rCpGms implicated in this analysis were annotated to chromatin states with gene regulatory functions, including active transcription start sites, flanking active transcription sites and enhancers (Fig. 4). Sixty percent of significant *cis-*rCpGm-gene pairs in the TCX had rCpGms annotated to transcription start sites and enhancer regions consistent with predicted regulatory functions of these rCpGm loci on gene expression (Supplementary Table 4).

We next hypothesized that *cis*-eQTMs where both the rCpGm and gene expression measures associate with the same AD endophenotype may implicate these *cis*-rCpGm-gene pairs in the biological mechanisms that may influence the endophenotype. We found 3664 FDR significant *cis*-rCpGm-gene-endophenotype triplets where the rCpGm and gene expression (GE) measures also significantly associated (FDR *P*-value ≤ 0.05) with the same endophenotype including total tau_TBS_, pTau_TX_, APOE_TX_ and pTau_FA_. We further reasoned that if these relationships were biologically meaningful, then the effect directions across all 3 models would be statistically concordant. For example, if the effect direction of the GE∼rCpGm association (model 1) is negative then the effect directions of the GE∼endophenotype (model 2) and rCpGm∼endophenotype (model 3) associations should be opposite, whereas a positive GE∼rCpGm association would translate to the same effect direction in models 2 and 3. We found that 99% (3644/3664) of the significant *cis*-rCpGm-gene-endophenotype triplets had concordant effect direction patterns, as we would expect if these relationships had biological underpinnings. We excluded the 20 discordant *cis*-rCpGm-gene-total tau_TBS_ triplets from downstream analyses. Of the 3644 concordant *cis*-rCpGm-gene-endophenotype triplets (*N* = 2931 unique *cis*-eQTM pairs), most were associated with total tau_TBS_ (*n* = 2594) or pTau_TX_ (*n* = 1018), where 698 significant *cis*-eQTMs were common to both endophenotypes and all with opposite directions of effect (Fig. 4). Of the remaining significant *cis*-eQTMs, 30 associated with APOE_TX_ and 2 with pTau_FA_.

Many of these *cis*-rCpGm-gene-endophenotype triplets implicate known AD and other neurodegenerative risk genes and loci. These include *ANKRD40* and *DNM2* that associate with APOE_TX_; *KLF16* and *SMAD3* with pTau_TX_; *APOC1, ANKRD40, C1QA, C1S, KNOP1, LRP1, NTN5, SPI1, TARDBP* and *TREM2* with total tau_TBS_, and *BIN1, CLU, HLA* genes, *MAF, PLEKHA1, TOMM40* and *ZCWPW1* with both total tau_TBS_ and pTau_TX._^2,43,64-67^ There were 6 eQTMs shared between total tau_TBS_ and APOE_TX_ (*ANKRD40, RP11-294J22.6, DGCR8, LINC01311, CRIPAK* and *NELFA*), 5 shared between total tau_TBS_, pTau_TX_ and APOE_TX_ (*ELOF1, PRKCSH, CNN1, CCDC159* and *CTD-2342J14.6*), 2 shared between total tau_TBS_, pTau_TX_ and pTau_FA_ (*ABCA17P* and *NTHL1*) and 2 shared between pTau_TX_ and APOE_TX_ (*LPPR2* and *RP11-79P5.9*). Together, these results suggest not only that the significant *cis*-eQTMs are biologically relevant but that these pairs may reflect an important regulatory system that underlies dysregulated AD gene expression networks, particularly those that relate to levels of tau proteins in the TCX.

We next investigated biological pathways enriched in the significant *cis*-rCpGm-gene-endophenotype triplet sets. We performed separate gene set enrichment analyses with genes from the significant triplet sets associated with total tau_TBS_ (1093 unique genes from 2594 associations), pTau_TX_ (671 unique genes from 1018 associations), APOE_TX_ (30 unique genes and associations), and both total tau_TBS_ and pTau_TX_ (464 unique genes from 698 associations). Only the total tau_TBS_ gene set showed enrichment with GO biological pathways relating to cell adhesion (Bonferroni *P*-value = 1.08E-4). We next investigated enrichment of cell-type marker genes defined by BRETIGEA^62^ for each gene set. We calculated enrichment using the top 100 and top 1000 BRETIGEA marker genes for each cell type. We found that oligodendrocyte marker genes were enriched in all gene sets, especially those associated with tau endophenotypes. Oligodendrocyte enrichment using top 100 and top 1000 BRETIGEA marker genes was as follows: APOE_TX_ (*P*-value: Top 100 = 0.15, Top 1000 = 4.9E-3), pTau_TX_ (*P*-value: Top 100 = 0.01, Top 1000 = 1.19E-07), total tau_TBS_ (*P*-value: Top 100 = 0.07, Top 1000 = 1.32E-08) and pTau_TX_ and total tau_TBS_ (*P*-value: Top 100 = 0.04, Top 1000 = 6.89E-06). Of the top 100 oligodendrocyte genes, *FAM222A* was implicated with the APOE_TX_ gene set, *KLK6, LDB3, MBP, CD22, TULP4, TMEM206, CCP110, PTPDC1* and *ASPA* with pTau_TX_, *PAIP2B, BCAS1, MBP, CD22, TULP4, PXK, CCP110, PTPDC1, PIEZO2* and *ASPA* with total tau_TBS_, as well as *MBP, CD22, TULP4, CCP110, PTPDC1* and *ASPA* with the overlapping pTau_TX_ and total tau_TBS_ gene set. This enrichment of oligodendrocyte marker genes suggests a potential link between brain tau levels, rCpGm, and dysregulation of oligodendrocyte genes.

### Genetic variation likely does not contribute to related CpGm, gene expression and tau variation

We next hypothesized that genetic variation may contribute to the variability of AD brain endophenotypes through its effects on the related gene expression and DNAm eQTMs identified above. We performed a *cis-*eQTL analysis and integrated findings from our previous *cis*-methylation QTL analysis (mQTL)^46^ and GWAS^20^ of these brain AD endophenotypes. We tested 45 872 850 TCX eQTLs between imputed variants ±500 kb from each gene identifying 976 368 (2.13%) that passed FDR correction (FDR *P*-value ≤ 0.05). To determine if genetic variants concurrently and significantly influence TCX rCpGm, gene expression, and AD endophenotypes, we integrated results from the brain AD endophenotype GWAS with our eQTL and mQTL findings. While no SNP reached FDR significance in both the eQTL and endophenotype GWAS, there were 11 unique SNPs from 40 mQTL-GWAS pairs that significantly associated (FDR *P*-value ≤ 0.05) with one or more of the following endophenotypes: Aβ40_TX_ (*N* = 1), Aβ40_FA_ (*N* = 22), Aβ40/42_FA_ (*N* = 6), APOE_TBS_ (*N* = 5) and APOE_FA_ (*N* = 6) (Supplementary Table 5). Six of these SNPs are located near the *APOE-NECTIN2-TOMM40-APOC1* locus (chr 19), three near *AP1S1-SERPINE1* (chr 7) and one each near the *GCSAML* and *ST3GAL1-ZFAT* loci. No SNPs associated significantly with all three measures of rCpGm, gene expression and AD endophenotype levels, suggesting that genetic variation likely does not contribute to related variation in CpGm, gene expression and tau levels.

### Proposed hypothetical model of dynamic brain gene expression changes in AD

**Figure 5:**
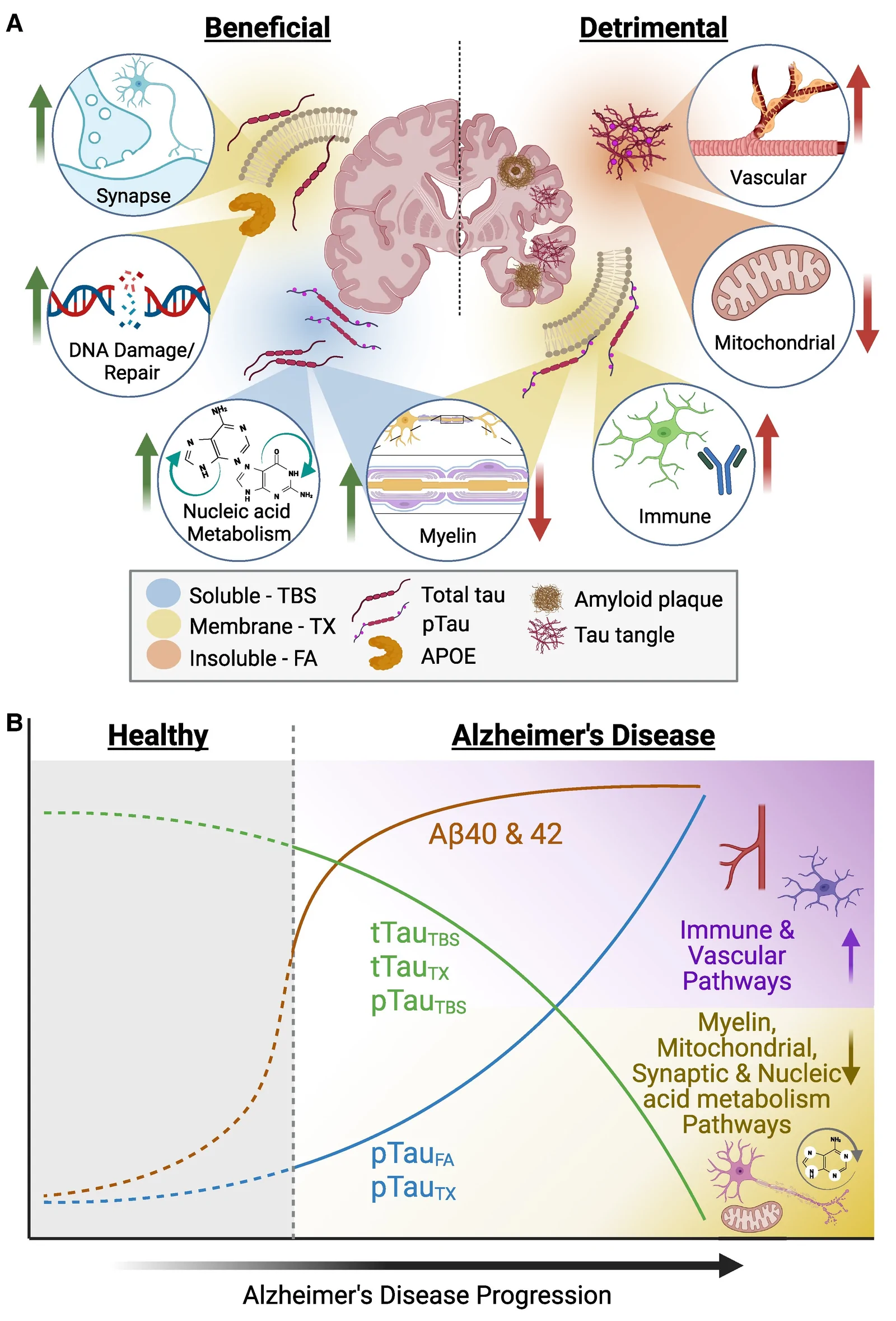
**Novel paradigm of dynamic changes in brain gene expression networks:** (**A**) Associations of AD brain protein species with transcriptional perturbations implicate specific dysregulated pathways and cell types. (**B**) Proposed dynamic model of gene expression changes linked to progression of proteostasis in the AD brain. Created in BioRender.com. Oatman, S. (2026) https://BioRender.com/39v2e4r. Aβ = amyloid-beta; FA = insoluble fraction; pTau = phospho-tau; TBS = soluble fraction; tTau = Total tau; TX = membrane fraction.

Using the collective findings from our study, we propose a novel hypothetical model of *dynamic brain gene expression changes* with progression of AD proteinopathy. Our proposed model is aligned with the hypothetical model of dynamic biomarker progression in AD,^21,68^ observed brain biochemical changes in hallmark AD proteins Aβ,^29,34-42^ tau,^24-33^ APOE^44,45^ and correlation of these biochemical changes with clinical progression of AD.^18,21^ With disease progression, the biochemical states of these proteins shift from soluble (TBS) and membrane-bound (TX) to insoluble (FA), based on these aforementioned studies and our data (Fig. 1). Furthermore, as brain total tau declines (tTau_TBS_, tTau_TX_), phosphorylated tau levels increase (pTau_FA_, pTau_TX_) with increasing AD neuropathology and risk (Fig. 1). Most of the AD brain transcriptome changes at both the single gene (Fig. 1A) and network levels (Fig. 2) are associated with brain biochemical tau levels, revealing inverse relationships between total tau and pTau. Importantly, the brain transcriptome perturbed with these AD proteins has strong enrichment of brain cell types (Figs 2 and 3) and biological processes (Fig. 2). Altogether, these findings support a model where a *beneficial (or neutral)* brain biochemical state of higher total tau and lower pTau is associated with increased levels of synaptic, DNA damage/repair, nucleic acid metabolism and myelin processes (Fig. 5A). In contrast, in a detrimental state of lower total tau and higher pTau, there is upregulation of vascular and immune, and downregulation of mitochondrial and myelin pathways (Fig. 5A). Our proposed model of *dynamic brain gene expression changes* (Fig. 5B) provides a cogent hypothesis that can be tested using longitudinal human cohorts, as well as model systems. If validated, these transcriptome changes that track with brain AD proteinopathy can pave the way for precision biomarkers that inform on the complex molecular perturbations occurring with AD progression.

## Discussion

Our study aimed to deconstruct the complexity of gene expression dysregulation in the AD brain by identifying transcriptional networks associated with individual proteins that are core to AD neuropathology. We identified gene expression changes associated with the biochemical state-specific levels of APOE, Aβ and tau in the AD brain. By layering additional omics measures of matched brain DNAm and genetic variant data, we were further able to identify regulatory epigenetic and genetic relationships of these brain AD endophenotype-specific gene expression changes.

We investigated transcriptome-wide associations for each of these brain AD endophenotypes with TCX gene expression measures obtained from 477 AD donors, respectively. We identified 5740 Bonferroni significant TWAS associations, 90% of which were with brain levels of tau including membrane-bound p-tau (pTau_TX_), insoluble p-tau (pTau_FA_) and soluble total tau (total tau_TBS_). Moreover, 42.4% of the brain AD endophenotype associations had nominally significant and congruent associations with AD diagnosis in an independent dataset from the same brain region.^57^ These results suggest that not only are there widespread changes in brain gene expression that occur with levels of specific tau biochemical states but that this dysregulation may be a component of the broader gene expression changes that are observed in AD brains compared to controls. Our results are consistent with a prior study,^17^ which identified epigenetic and corresponding transcriptomic changes in the AD brain associated with phosphorylated tau tangle burden but not with Aβ plaques. This broad transcriptional dysregulation that occurs with brain tau accumulation has also been observed in AD mouse models.^69,70^ We similarly did not observe widespread gene expression dysregulation associated with Aβ levels, which may suggest that tau, but not Aβ, underlies brain transcriptional dysregulation in AD. However, it should be noted that brain pTau and NFT accumulate in later stages of AD^21,68^; therefore, transcriptional perturbations that may associate with Aβ accumulation in earlier disease stages may not be captured here. Future studies that investigate gene expression changes in relation to brain tau and Aβ levels across the time course of AD will be important.

Our deep brain endophenotype TWAS approach enables dissection of brain transcripts and expression networks that are associated not only with specific core AD proteins but their individual biochemical fractions, as well as AD diagnosis and neuropathology. We found strong positive correlations in the directions of association for these 5740 TCX genes with insoluble AD protein fractions, AD diagnosis and neuropathology. In contrast, TWAS associations with brain levels of soluble pTau, total tau, APOE and membrane-bound total tau have negative directions to those with AD diagnosis and neuropathology. These results provide insights into the potentially dynamic brain gene expression changes that may ensue with shifts from soluble to insoluble biochemical states, particularly of tau species with progression of AD (Fig. 5).

We uncovered biological pathways, co-expression networks and cell types that may be specifically affected by these AD protein species (Fig. 5A). Genes and MEs that are down-regulated with total tau_TBS_ and conversely upregulated with pTau_TX_ were enriched for immune response related pathways and microglial genes. In contrast, genes upregulated with higher brain total tau_TBS_ and downregulated with pTau_TX_ were enriched for nucleic acid metabolism related pathways. Mitochondrial pathways are down and vascular pathways are up with pTau_FA_. Synaptic signalling and DNA damage response pathways are up with total tau_TX_ and APOE_TX_, respectively. While some of these pathways have previously been associated with AD diagnosis, our study begins to illuminate potential molecular mechanisms that are involved with specific aspects of AD-related proteostasis and their potential temporal progression. Our findings support a model where soluble and membrane-bound states of tau and APOE promote integrity of synaptic signalling and DNA metabolism (Fig. 5A). With increasing pTau levels, there is increasing brain immune response, aberrant vascularization and dampened mitochondrial function. Given that these findings are based on associations from an AD only dataset, the temporality of this proposed progression needs to be functionally validated in model systems and across the spectrum of AD in longitudinal human cohorts. Furthermore, the causal direction, i.e. AD protein disrupting gene expression versus transcriptional perturbation causing dysproteostasis versus bidirectional effects, needs to be investigated in experimental models. Nevertheless, our results suggest a hypothetical model of *dynamic brain gene expression changes* with progression of AD, akin to the hypothetical model of dynamic biomarker changes in AD^21,68^ (Fig. 5B).

Additionally, our TWAS results implicate specific brain cell-type proportions associated with the AD endophenotype. Proportions of astrocytes, microglia, and endothelial cells showed more specific and stronger associations with brain tau levels, suggesting that these cell types may have higher vulnerabilities to tau than amyloid in the TCX. Neuronal and oligodendrocytic proportions had broader and generally opposite directions of associations with these endophenotypes. We note, however, that the cell-type proportions used in this study are estimates based on bulk deconvolution methods. Future studies of single-cell data and human iPSC-derived models of different brain cell types can validate our findings of cell type–specific vulnerabilities to different AD protein species. Importantly, the majority of our TWAS results remain significant even after adjusting for estimated neuronal proportions. This suggests that while the brain AD endophenotypes may have differential effects on proportions and/or gene expression of certain cell types, many of the TWAS findings are not a consequence of brain cell proportion changes in AD.

One potential mechanism that may lead to variability of these biochemical AD protein species is genetic and/or epigenetic variability that influences brain gene expression and endophenotypes levels. To uncover potential regulatory mechanisms that may explain our TWAS findings, we integrated brain gene expression and endophenotype data with measures of DNAm^46^ and genetic variants^20^ obtained from the same brain donors. There are 12 973 *cis*-eQTMs where regional DNAm (rCpGm) levels significantly associate with expression levels of nearby genes. Many of these rCpGms reside in transcriptionally active sites supporting their predicted regulatory function. Interestingly, about a third of these significant *cis*-rCpGm-gene pairs (3644/12 973) also associated significantly with brain AD endophenotypes in a biologically congruent manner. Many of these *cis*-rCpGm-gene-endophenotype associations were with levels of soluble total tau (total tau_TBS_) and membrane-bound pTau (pTau_TX_) with the rest involving APOE_TX_ and insoluble pTau (pTau_FA_). Notably, a sizable subset of these *cis*-rCpGm-gene pairs associated with both total tau_TBS_ and pTau_TX,_ all with opposite directions of effect. Importantly, in all the eQTMs related to tau or APOE, we found enrichment of oligodendrocyte marker genes. These findings suggest that DNAm variability is likely an important mechanism regulating the gene expression networks we identified in the TCX relating to brain tau and to some extent APOE levels. This epigenetic mechanism appears to preferentially affect oligodendrocyte gene expression, which is consistent with findings from our^1,5,46,71^ and other groups^72^ that demonstrated oligodendrocyte/myelin expression network disruptions with AD and other tauopathies (Fig. 5). While these findings cannot unequivocally determine whether tau is a cause or consequence (or both) of oligodendrocyte damage in AD, they demonstrate the role of epigenetic changes in driving transcriptional disruptions for this cell-type in a tau-related manner.

Since regulatory genetic variants that associate with brain gene expression and DNAm levels can provide information on causal direction, we sought to identify eQTLs and mQTLs that also influence brain AD endophenotypes. We found there were no eQTLs that also associated with brain AD endophenotypes and only 40 such mQTLs, most of which clustered around the *APOE* locus and associated with brain Aβ and APOE, but not tau levels. Collectively, these findings indicate that with some exceptions, *cis-*regulatory variants are not a major driver of the vast transcriptional changes that occur with changes in brain levels of AD-related proteins. Rather, *cis-*epigenetic changes that relate particularly to changing brain tau levels, or a yet unknown mechanism that influences both, could be regulating some of these transcriptional perturbations.

Our study has many strengths, including a large cohort of AD donors characterized with multiple layers of information such as neuropathology, gene expression, DNAm, genetic variants and precise brain endophenotypes obtained from TCX for different biochemical states of core AD proteins. The availability and depth of multi-omics and brain endophenotype data from the same brain region and proximal sections increases the corresponding rigour of the associations we identified. The presence of genetic and epigenetic data enables the interrogation of potential regulatory mechanisms underlying the endophenotype associations with brain transcriptional perturbations. Our multitude of analytic approaches, including TWAS and co-expression networks, combined with enrichment for biological pathways and cell types, yields converging evidence of disruptions in cell-specific pathways, with particular fractions of tau. Altogether, our work sets the stage for a novel hypothetical paradigm of *dynamic changes in brain gene expression networks* that parallel the temporal shifts in biochemical states of core AD-proteins (Fig. 5), particularly that of tau species from soluble total and pTau to membrane-bound and insoluble pTau.

There are also some limitations to our study. These findings are based on measures taken from postmortem tissue reflecting a single point at advanced, end-stage disease. Hence our proposed hypothetical paradigm of *dynamic brain gene expression-endophenotype changes* in AD needs to be validated potentially in human iPSC-derived models or in future antemortem human cohorts with collection of longitudinal multi-omics measures from peripheral tissues alongside brain biomarkers that parallel changes in these biochemical species of AD proteins. Future experimental studies should also investigate the causal relationship between the different biochemical species, especially total tau and pTau. While these measures are correlated, they are thought to reflect different aspects of AD biology, with total tau indicative of neuronal damage that may also be present in non-AD and pTau indicative of AD-related disease progression.^21,73^ Indeed, both total tau and pTau levels are utilized as CSF biomarkers of AD.^21,68,73^ Given this, we opted to include both Total tau and pTau related results, as they may reflect correlated but distinct aspects of AD.

Additionally, the gene expression and biochemical measures were collected from the TCX of a neuropathologically diagnosed AD cohort with Braak stages IV-VI. As such, it will be important for future studies to evaluate the generalizability of these dynamic gene expression and AD protein changes across the full range of AD pathology, including the earliest pathologic stages, and in other brain regions.

Another potential caveat is that our transcriptome and methylome data were generated from bulk brain tissue. Although these bulk tissue measures are able to capture global changes, studies have shown that different cell types have distinct gene expression association patterns with Aβ and tau.^74^ As such, it will be important to investigate these gene networks on a single cell type or single cell/nuclei level to parse out the role individual cells play in these dysregulated gene networks. Nevertheless, our enrichment analyses uncovered pathways perturbed in specific cell types and their relationship with particular AD endophenotype measures. Moreover, our sensitivity analyses determined that our associations are not driven by neuronal proportion changes, AD neuropathology measured by Braak stage and Thal phase, or gene expression outliers. Additionally, our three model pairwise framework investigating co-variation between DNA methylation, gene expression, and the AD proteins does not incorporate other known regulatory mechanisms that could influence these relationships, such as trans-regulatory elements, local chromatin structure, transcription factor binding or non-coding RNAs, as we did not have these measures available in our study. Future investigation of the impact of these additional regulatory elements will be important in teasing apart the direction of causality in these associations. Our cohort comprises only non-Hispanic White (NHW) donors so it will be important to replicate our findings in future studies of donors from different ancestral backgrounds. Finally, as is common in AD brains, donors in our cohort often have co-pathologies such as TDP-43 or Lewy body deposits which may increase the heterogeneity of our dataset. Nevertheless, the consistency and biological congruence of our findings suggest that these *brain gene expression-endophenotype changes* are robust to the heterogeneity in the neuropathological co-pathologies inherent to AD brains.

In summary, our study investigated brain transcriptome changes that associate with changing brain levels of proteins core to AD pathophysiology and their specific biochemical states. We identified widespread brain gene expression dysregulation associated with changes in the AD brain, especially with levels of brain tau. Associated transcripts and their networks implicated pathway and brain cell type perturbations that are linked to specific tau species. These tau related transcriptional dysregulations are congruent with AD neuropathology and diagnostic associations both in this and independent datasets, underscoring their relevance to the disease process. While genetic variation likely does not play a major causative role in these dysregulations, we linked epigenetic variation in DNAm to oligodendrocyte/myelin gene expression perturbations and the latter to variable brain tau endophenotypes. Collectively, these findings begin to illuminate potential cellular and molecular perturbations that are related with changes in specific brain biochemical states of hallmark AD proteins. Importantly, they support a novel hypothetical model of *dynamic changes in brain gene expression networks* that are parallel to these shifts in AD proteins in the AD brain (Fig. 5). This model, if validated in longitudinal human and experimental models, can inform on specific, dynamic molecular disruptions and therefore hold the potential as dynamic, precision biomarkers of brain AD progression.

## Supplementary Material

fcag326_Supplementary_Data

## Data Availability

The data in this manuscript, including full results for TWAS, gene enrichment analysis and multi-omics integration, are available via the AD Knowledge Portal (https://adknowledgeportal.synapse.org). The AD Knowledge Portal is a platform for accessing data, analyses and tools generated by the Accelerating Medicines Partnership (AMP-AD) Target Discovery Program and other National Institute on Aging (NIA)-supported programs to enable open- science practices and accelerate translational learning. The data, analyses and tools are shared early in the research cycle without a publication embargo on secondary use. Data are available for general research use according to the following requirements for data access and data attribution (https://adknowledgeportal.synapse.org/DataAccess/Instructions). For access to content described in this manuscript see the manuscript landing page: https://www.synapse.org/Synapse:syn64679963/datasets/.

## Code availability

Code used in this manuscript for data analyses can be accessed via the AD Knowledge Portal: https://www.synapse.org/Synapse:syn75046458.
