# Supplementary material for: Brain multiomic profiling identifies tau-related transcriptomic dysregulation in Alzheimer’s disease
Source: Brain Commun. 2026 Aug 27;8(5):fcag326. doi: 10.1093/braincomms/fcag326 (PMC13550776; doi:10.1093/braincomms/fcag326)
Supplement: Supplementary file 1 [file fcag326_supplementary_data.pdf]

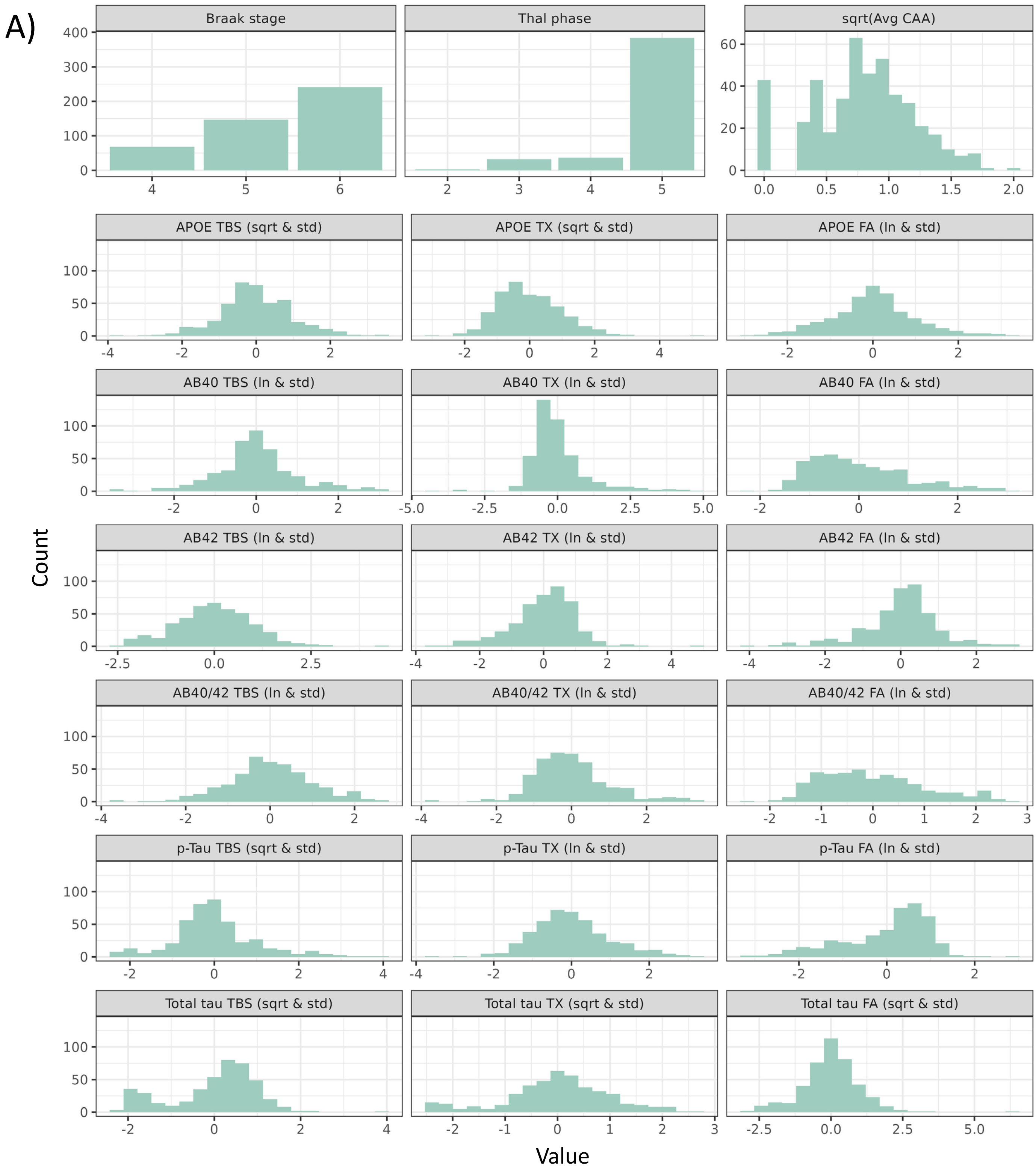

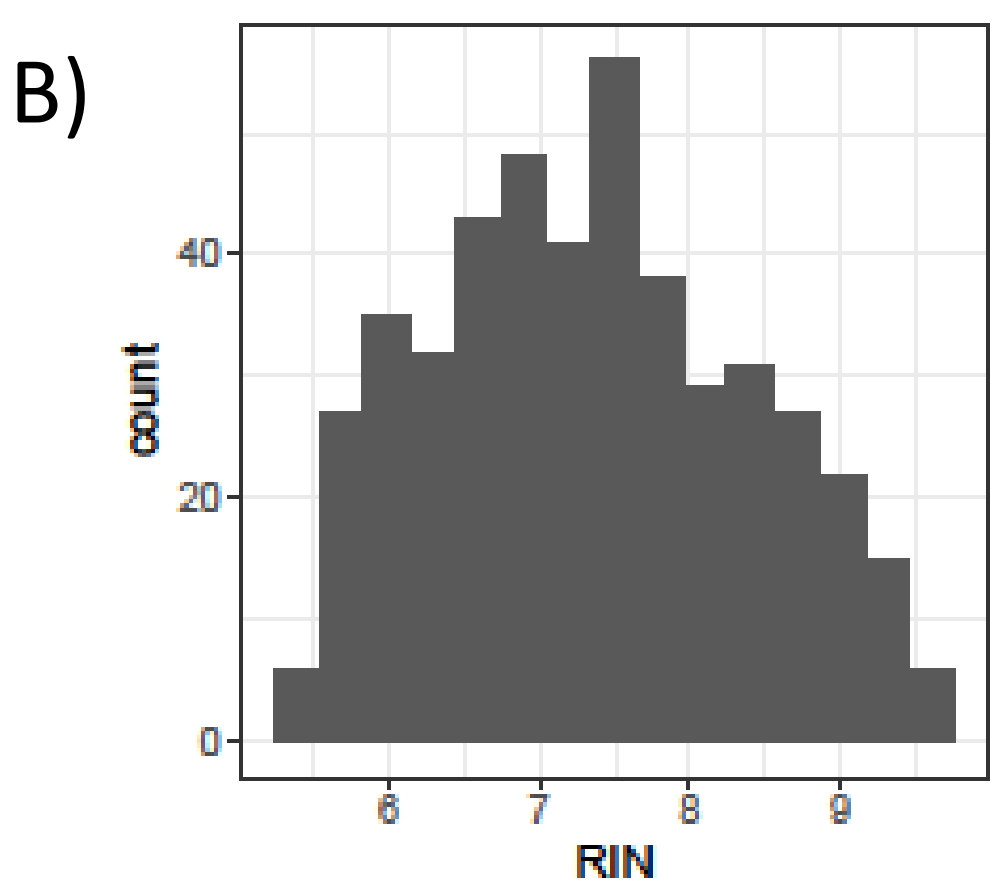

Supplementary Figure 1: Histograms of AD endophenotypes used in each transcriptome-wide association study (TWAS).

(A) Histogram plots of AD endophenotypes for each brain tissue type. Biochemical measures were either natural log or square root transformed to approximate a normal distribution and then standardized. (B) Histogram of RIN scores across gene expression samples.

AB = Amyloid beta, **CAA** = Cerebral amyloid angiopathy**,** **FA**= insoluble formic acid tissue fraction, **ln** = natural log transformed, **p-Tau** = phospho-tau, **RIN** = RNA integrity number, **sqrt** = square root transformed, **std** = standardized, **TBS** = soluble tris-buffered saline tissue fraction, **TCX** = Temporal Cortex, **TX**= membrane associated Triton-X tissue fraction

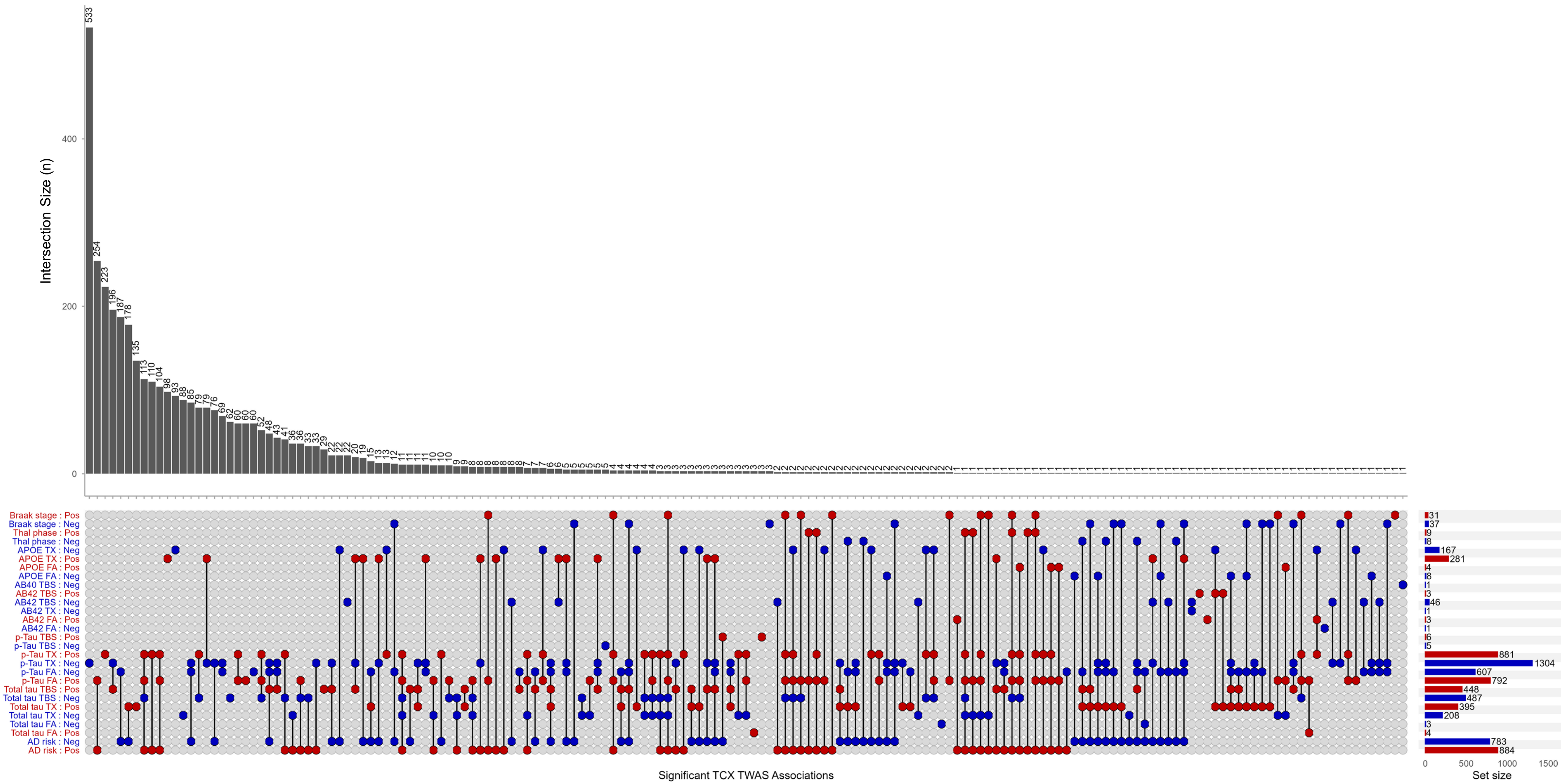

Supplementary Figure 2: Extended Upset plot of significant temporal cortex TWAS associations.

Upset plot showing all Bonferroni significant gene (Bonferroni *pval* ≤ 0.05) expression associations across the TWAS for each endophenotype measured using linear regression including the total intersection set numbers and the intersections for each phenotype and nominal AD risk associations (*pval* ≤ 0.05) from an independent dataset.

AB = Amyloid-beta, **AD** = Alzheimer's Disease, **FA** = Insoluble formic acid fraction; **GE** = Gene Expression; **N** = Number; **Neg** = negative; **pos** = positive; **pTau** = phospho-tau; **TBS** = soluble tris buffered saline fraction; **tTau** = total tau; **TWAS** = Transcriptome-wide association study, **TX**= membrane associated Triton-X fraction

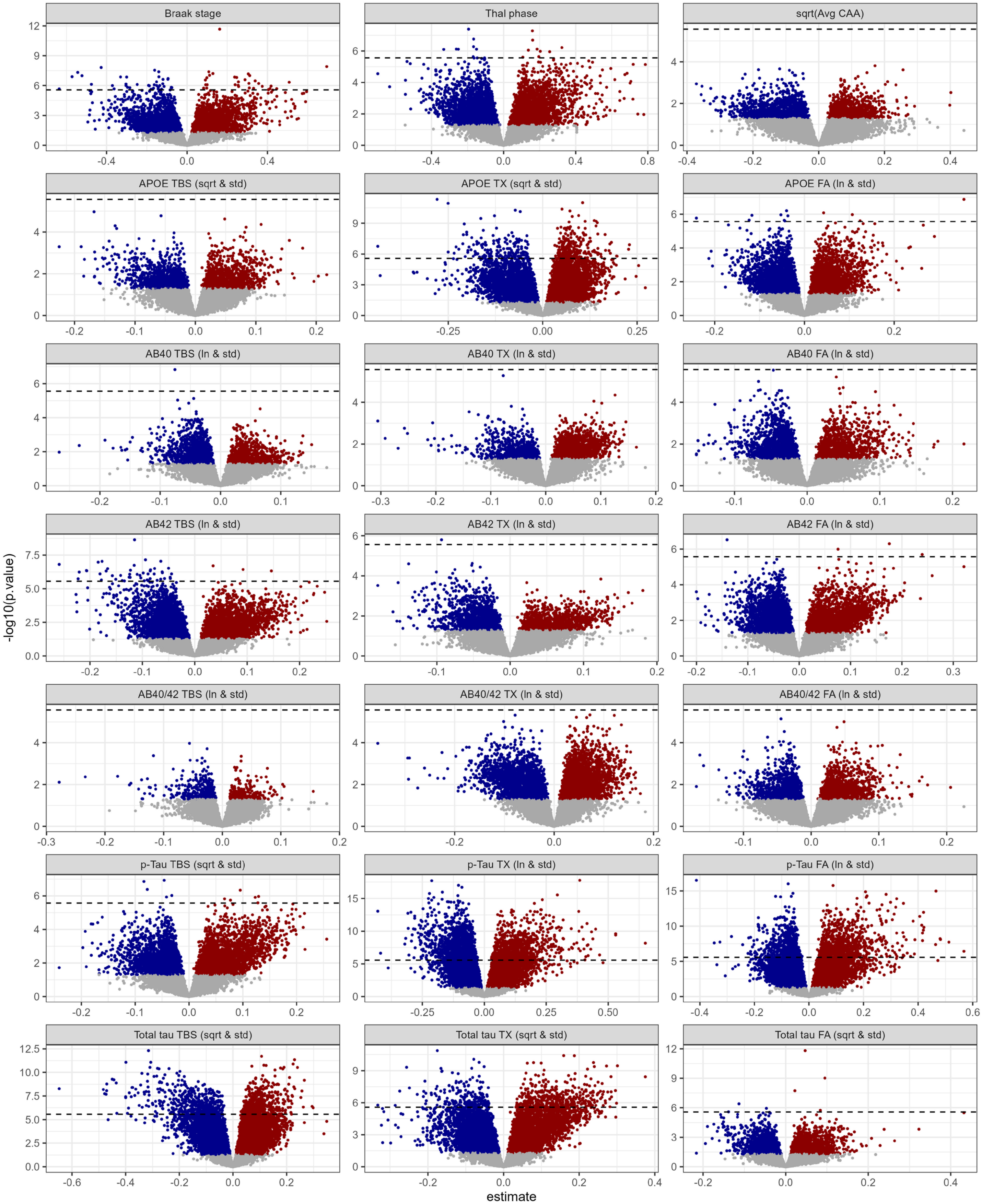

Supplementary Figure 3: Volcano plots of TWAS for each endophenotype investigated.
Volcano Plots of the TWAS linear regression results for each endophenotype tested. Blue dots are genes with a negative estimate and p-value <= 0.05, red dots are genes with a positive estimate and p-value <= 0.05, grey dots are genes with a p-value > 0.05. The black dashed line indicates the Bonferroni significance threshold of p-value = 2.72E-06.
AB = Amyloid beta, **FA**= insoluble formic acid tissue fraction, **ln** = natural log transformed, **p-Tau** = phospho-tau, **sqrt** = square root transformed, **std** = standardized, **TBS** = soluble tris-buffered saline tissue fraction, **TWAS** = Transcriptome-wide association study, **TX**= membrane associated Triton-X tissue fraction

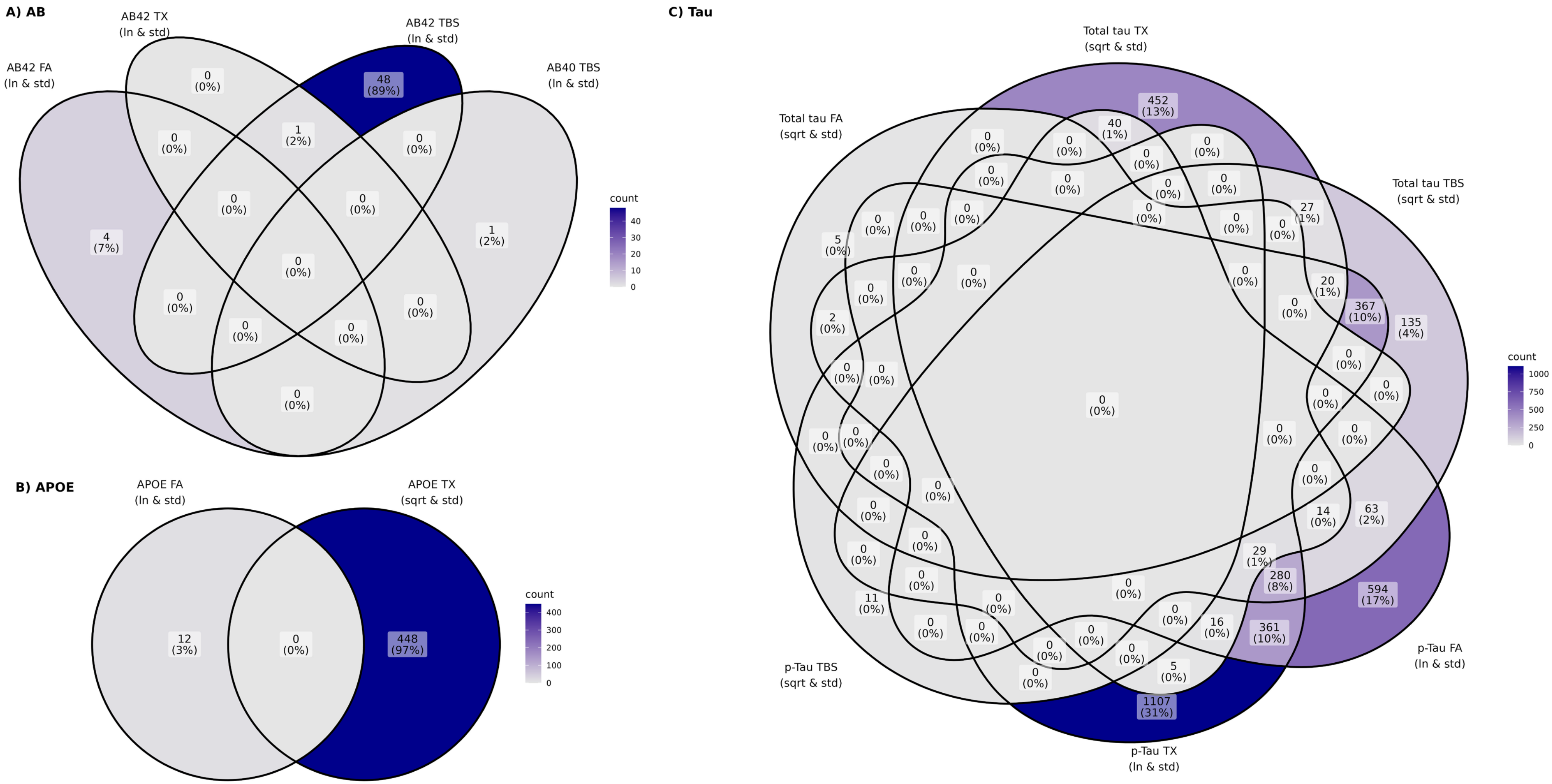

Supplementary Figure 4: Overlap of significant temporal cortex gene expression associations across tissue fractions of same proteins.

Venn diagrams depicting number of Bonferroni significant (Bonferroni pval ≤ 0.05) temporal cortex gene expression associations from the primary TWAS using linear regression across tissue fractions of amyloid, APOE and tau related proteins. Count denotes the number of genes which are associated with indicated protein and tissue fraction.

AB = amyloid beta, **FA** = Insoluble formic acid tissue fraction, **ln** = natural log transformed, **p-tau** = phospho-tau, **sqrt** = square root transformed, **std** = standardized, **TBS** = soluble tris buffered saline tissue fraction; **TX**= membrane associated Triton-X tissue fraction

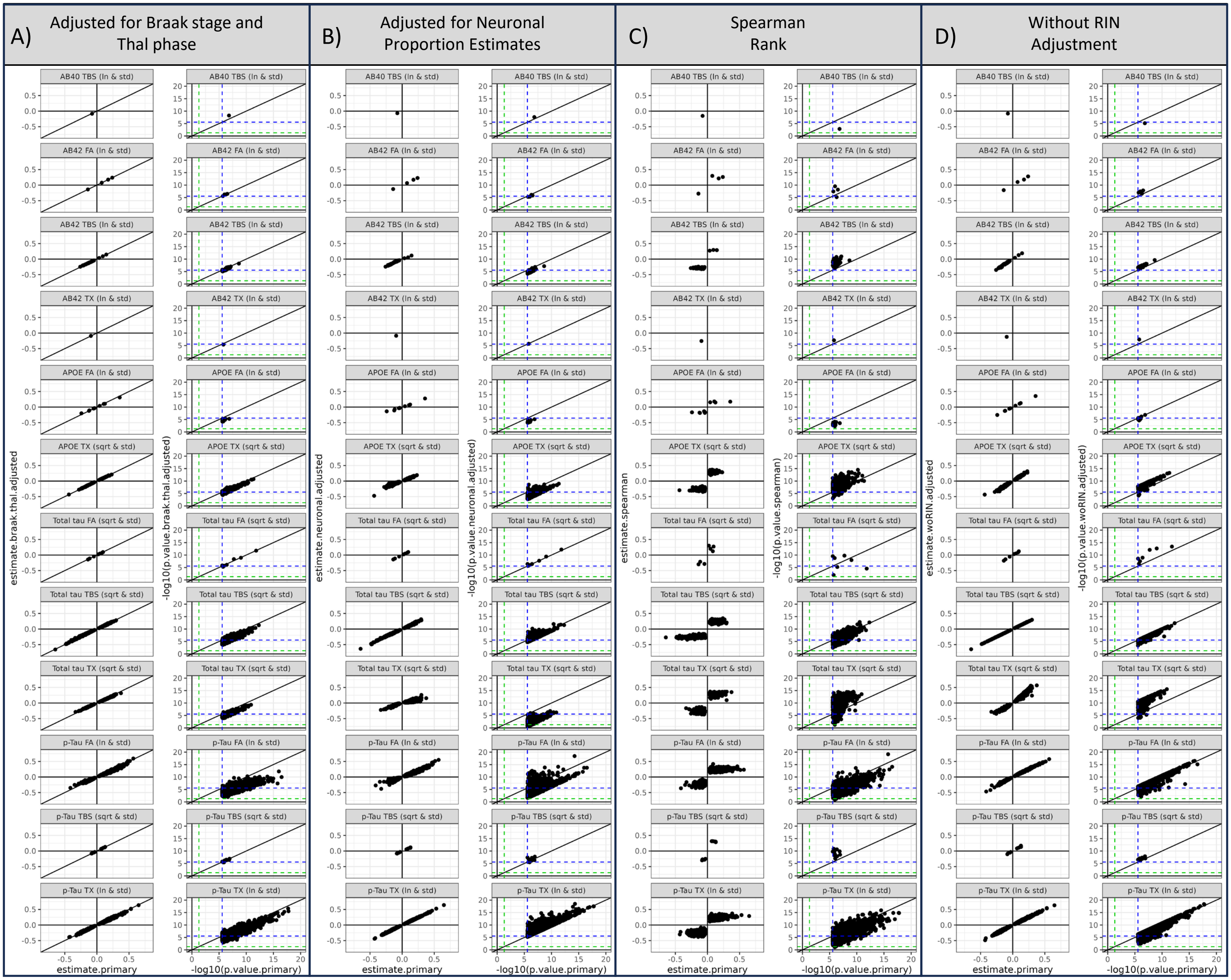

E)

Phenotype in TWAS	Primary TWAS	Braak & Thal adjusted		Spearman rank test		Neuronal proportion adjusted		without RIN	
	N Bonf sig	N Bonf sig	N Nom sig	N Bonf sig	N Nom sig	N Bonf sig	N Nom sig	N Bonf sig	N Nom sig
AB40 TBS	1	1	1	0	1	1	1	0	1
AB42 FA	4	4	4	3	4	2	4	4	4
AB42 TBS	49	32	49	49	49	13	49	49	49
AB42 TX	1	0	1	1	1	1	1	1	1
APOE FA	12	0	12	0	12	0	12	6	12
APOE TX	448	290	448	418	448	104	448	443	448
Total tau FA	7	6	7	4	7	7	7	7	7
Total tau TBS	935	543	935	640	935	698	935	734	935
Total tau TX	603	192	603	560	602	11	543	594	603
p-Tau FA	1399	439	1399	674	1397	566	1399	1225	1399
p-Tau TBS	11	8	11	11	11	10	11	11	11
p-Tau TX	2185	1542	2185	1373	2184	2083	2185	1934	2185
Total	***5655***	***3057***	***5655***	***3733***	***5651***	***3496***	***5595***	***5008***	***5655***

Supplementary Figure 5: Comparison of primary and sensitivity TCX TWAS model results for Bonferroni significant genes in the primary TWAS models.

Comparison of Bonferroni significant biochemical measure TWAS test statistics from the TCX primary and sensitivity linear regression models. Sensitivity models included adjusting for Braak stage and Thal phase (A), adjusting for neuronal cell type proportion estimates (B), non-parametric Spearman rank tests (C), and without including RIN as a covariate (D). A-D) Primary model statistics are on the x-axes while sensitivity model statistics are on the y- axes. Blue dashed lines denote the TCX Bonferroni p-value = 2.719E-06 and green dashed lines denote p-value = 0.05. E) Table depicts the number of Bonferroni (p-value ≤ 2.719E-06) and nominally (p-value ≤ 0.05) significant results for each sensitivity analysis.

Bonf = Bonferroni, **FA**= insoluble formic acid tissue fraction, **ln** = natural log transformed, **Nom** = nominally, **p-Tau** = phospho-tau, **sig** = significant, **sqrt** = square root transformed, **std** = standardized, **TBS** = soluble tris-buffered saline tissue fraction, **TCX** = Temporal Cortex, **t-Tau** = Total tau; **TWAS** = transcriptome-wide association study, **TX**= membrane associated Triton-X tissue fraction, **woRIN** = without RNA Integrity Number

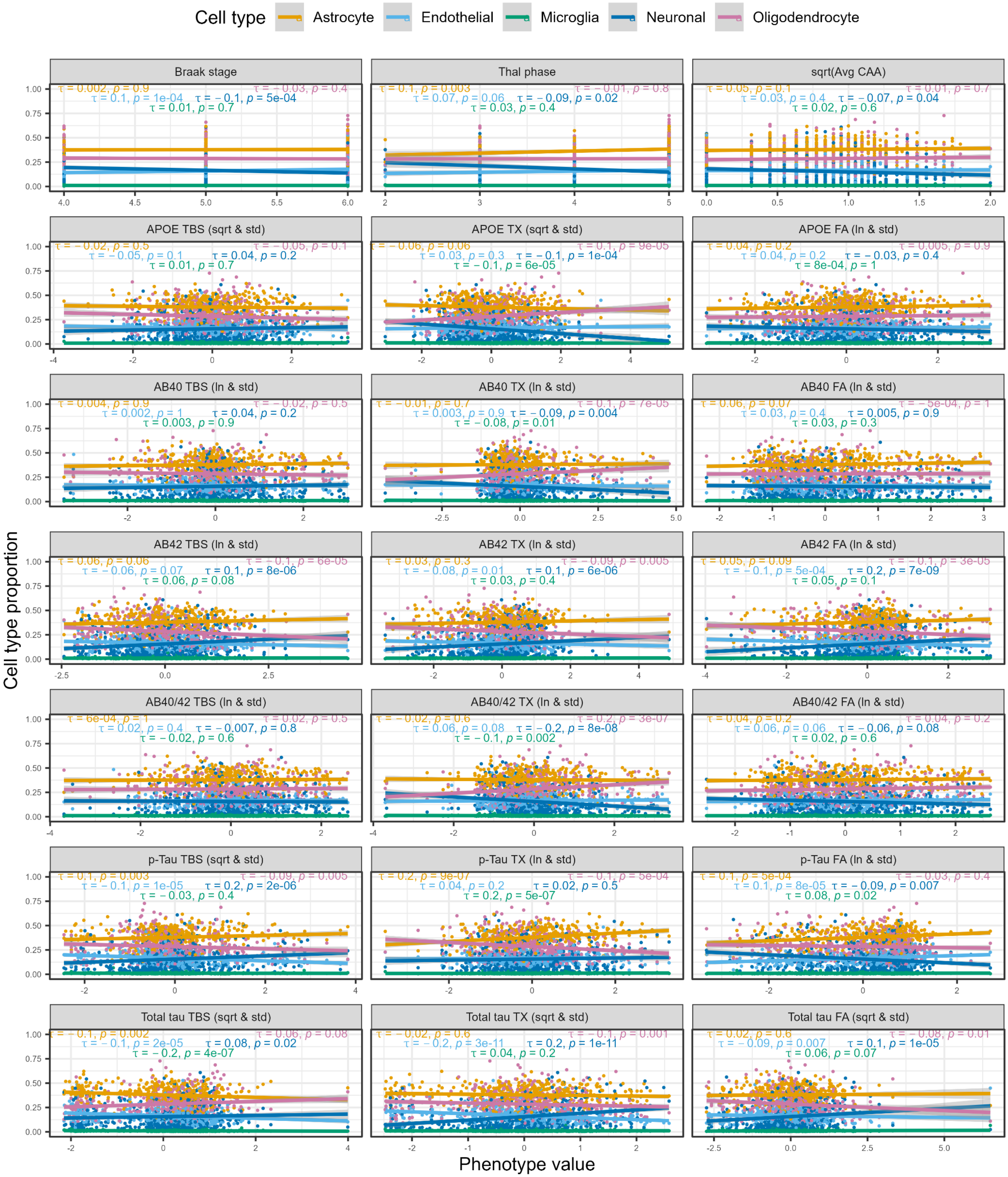

Supplementary Figure 6: Estimated cell type proportions across each cell type and phenotype.

Dot plot of estimated cell type proportions across each phenotype and cell type. Each dot represents the estimated cell type specific proportion of a single sample. Trend lines represent the linear relationship (y~x) between estimated cell type proportion and phenotype for each major cell type. Statistics in each plot represent the non-parametric Kendall's rank correlation statistics between each cell type and phenotype including Kendall's τ and p-value. Full statistics are available in supplemental table 3. Colors represent each cell type.

AB = Amyloid-beta, **FA** = Insoluble formic acid tissue fraction, **ln** = natural log, **pTau** = phospho-tau, **std** = standardized, **sqrt** = square root, **TBS** = soluble tris buffered saline tissue fraction, **TX**= membrane associated Triton-X tissue fraction

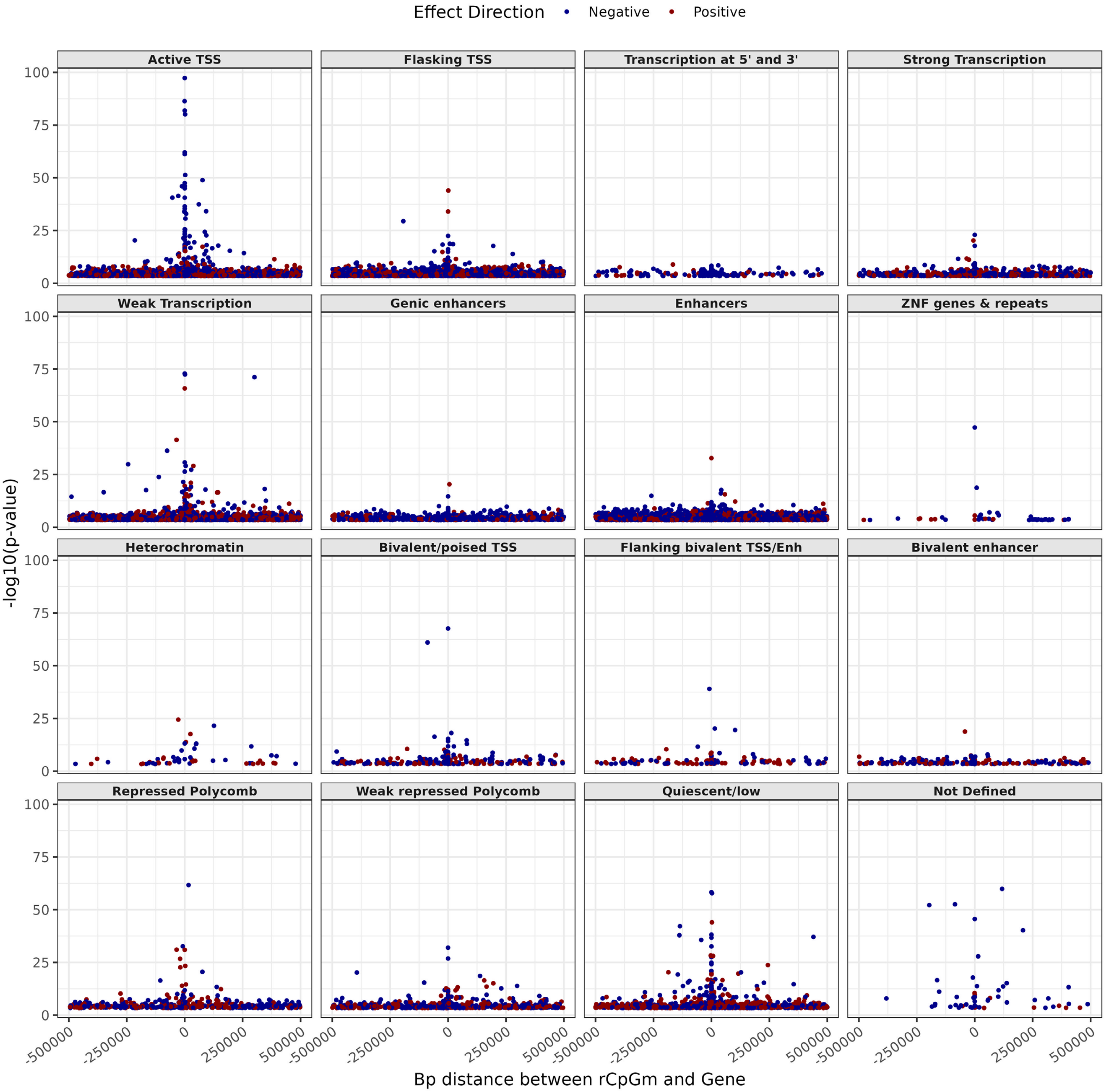

Supplementary Figure 7: Characterization of the eQTM associations by rCpGm chromatin state annotation.
Dot plot of FDR significant cis-eQTMs in the TCX by the predicted annotation of the rCpGm. Each dot is a single eQTM association. Red indicates positive and blue indicates negative effect directions. The x-axis is the shortest distance between the tested rCpGm and gene in base pairs (bp), a negative bp distance indicates the rCpGm is before the gene and a positive value indicates the rCpGm is after the gene reading the genome from 5' to 3' on the forward strand.
Bp = base pair, **Enh** = Enhancer, **TCX** = Temporal Cortex, **TSS**= Transcription start site, **ZNF** = Zinc finger

Supplementary Table 1: Demographics of Dataset. Summary demographics of the transcriptomic datasets analyzed after quality control.
CAA=Cerebral amyloid angiopathy, N= Number, SD = Standard Deviation

		All	*APOE ε 4* positive	*APOEε4* negative	Males	Females
**Total N**		456	307	149	215	241
**N: Sex**	**Male**	215	145	70	215	0
	**Female**	241	162	79	0	241
**N: *APOEε4* dose**	**0**	149	0	149	70	79
	**1**	245	245	0	113	132
	**2**	62	62	0	32	30
**Mean age at death**		80.21	80.66	79.28	78.6	81.66
**SD age at death**		9.1	8.704	9.832	8.796	9.142
**N: Braak stage**	**4**	68	47	21	40	28
	**5**	147	87	60	76	71
	**6**	241	173	68	99	142
**N: Thal phase**	**2**	3	1	2	1	2
	**3**	32	15	17	18	14
	**4**	37	27	10	16	21
	**5**	384	264	120	180	204
**Mean CAA**		0.815	0.910	0.617	0.905	0.734
**SD Mean CAA**		0.680	0.703	0.583	0.689	0.663
**N: Average CAA**	**0-<1**	296	190	106	129	167
	**1-<2**	123	85	38	67	56
	**2-<3**	32	27	5	16	16
	**3-4**	5	5	0	3	2

Supplementary Table 2: Enrichment of Cell Type Marker genes for significant TCX TWAS Results. Significant gene expression associations from the TCX TWAS were tested for enrichment of cell type marker genes defined by BRETIGEA (see methods for details). Only test sets with >5 significant genes were tested using a hypergeometric test. Enrichment using the top 100 and all 1000 marker genes are shown. The total gene population is defined as all autosomal genes reliably detected above background (N=18,383). Test sets are defined as the gene associated with the phenotype in a positive or negative effect direction. Bonferroni p-values were calculated for the 1000 (N tests = 63) and 100 (N tests = 20) marker gene sets separately.
AB =Amyloid-beta, FA= insoluble formic tissue fraction, ln = natural log transformed, N = Number of genes present in Test set, neg = negative, p-Tau = phospho-tau, pos = positive, sqrt = square root transformed, std = standardized, TBS = soluble tris-buffered saline tissue fraction, TCX = Temporal Cortex, t-Tau = Total tau, TX= membrane associated Triton-X tissue fraction

			Using all 1000 Marker genes			Using Top 100 Marker genes		
**Test Set**	**Cell Type**	**Total genes in Test Set**	**N**	**p-value**	**Bonferroni p-value**	**N**	**p-value**	**Bonferroni p-value**
Total tau TX (sqrt & std):pos	Neuronal	395	111	2.70E-49	1.70E-47	15	3.55E-09	7.10E-08
p-Tau TX (ln & std):pos	Astrocyte	881	163	1.15E-45	7.22E-44	35	1.91E-21	3.82E-20
Total tau TBS (sqrt & std):neg	Microglia	487	89	1.74E-24	1.10E-22	22	1.38E-14	2.76E-13
Total tau TBS (sqrt & std):neg	Endothelial	487	80	3.89E-19	2.45E-17	13	2.35E-06	4.70E-05
p-Tau FA (ln & std):pos	Astrocyte	792	103	8.34E-17	5.25E-15	16	5.51E-06	1.10E-04
p-Tau FA (ln & std):pos	Endothelial	792	103	8.34E-17	5.25E-15	12	1.22E-03	2.44E-02
Total tau TX (sqrt & std):neg	Endothelial	208	34	8.20E-09	5.17E-07	5	5.58E-03	1.12E-01
AB42 TBS (ln & std):neg	Oligodendrocyte	46	11	2.69E-05	1.69E-03	1	2.22E-01	1.00E+00
p-Tau TX (ln & std):pos	Microglia	881	73	2.13E-04	1.34E-02	19	2.36E-07	4.71E-06
APOE TX (sqrt & std):neg	Neuronal	167	16	1.99E-02	1.00E+00	0	-	-
p-Tau FA (ln & std):neg	Neuronal	607	39	1.59E-01	1.00E+00	0	-	-
Braak stage:pos	Endothelial	31	3	2.37E-01	1.00E+00	0	-	-
p-Tau TBS (sqrt & std):pos	Astrocyte	6	1	2.85E-01	1.00E+00	0	-	-
p-Tau FA (ln & std):pos	Microglia	792	47	2.87E-01	1.00E+00	0	-	-
APOE FA (ln & std):neg	Neuronal	8	1	3.61E-01	1.00E+00	0	-	-
Thal phase:neg	Neuronal	8	1	3.61E-01	1.00E+00	0	-	-
Thal phase:pos	Astrocyte	9	1	3.96E-01	1.00E+00	1	4.79E-02	9.58E-01
Thal phase:pos	Endothelial	9	1	3.96E-01	1.00E+00	0	-	-
APOE TX (sqrt & std):neg	Microglia	167	10	4.24E-01	1.00E+00	1	6.00E-01	1.00E+00
Braak stage:neg	Neuronal	37	2	6.05E-01	1.00E+00	0	-	-
APOE TX (sqrt & std):pos	Oligodendrocyte	281	14	6.71E-01	1.00E+00	1	7.87E-01	1.00E+00
Total tau TX (sqrt & std):neg	Astrocyte	208	10	7.01E-01	1.00E+00	0	-	-
p-Tau TX (ln & std):pos	Endothelial	881	44	7.46E-01	1.00E+00	7	2.03E-01	1.00E+00
Braak stage:pos	Astrocyte	31	1	8.24E-01	1.00E+00	0	-	-
Braak stage:pos	Microglia	31	1	8.24E-01	1.00E+00	0	-	-
Braak stage:pos	Neuronal	31	1	8.24E-01	1.00E+00	0	-	-
Braak stage:neg	Astrocyte	37	1	8.74E-01	1.00E+00	0	-	-
Braak stage:neg	Endothelial	37	1	8.74E-01	1.00E+00	0	-	-
Total tau TBS (sqrt & std):neg	Astrocyte	487	21	8.91E-01	1.00E+00	0	-	-
AB42 TBS (ln & std):neg	Astrocyte	46	1	9.24E-01	1.00E+00	0	-	-
AB42 TBS (ln & std):neg	Endothelial	46	1	9.24E-01	1.00E+00	0	-	-
APOE TX (sqrt & std):neg	Astrocyte	167	3	9.95E-01	1.00E+00	0	-	-
APOE TX (sqrt & std):neg	Endothelial	167	3	9.95E-01	1.00E+00	0	-	-
Total tau TX (sqrt & std):neg	Microglia	208	4	9.97E-01	1.00E+00	0	-	-
Total tau TX (sqrt & std):neg	Oligodendrocyte	208	4	9.97E-01	1.00E+00	0	-	-
p-Tau TX (ln & std):neg	Oligodendrocyte	1304	50	9.98E-01	1.00E+00	3	9.76E-01	1.00E+00
Total tau TBS (sqrt & std):pos	Neuronal	448	12	9.99E-01	1.00E+00	0	-	-
Total tau TBS (sqrt & std):pos	Oligodendrocyte	448	12	9.99E-01	1.00E+00	1	9.16E-01	1.00E+00
APOE TX (sqrt & std):neg	Oligodendrocyte	167	2	9.99E-01	1.00E+00	2	2.30E-01	1.00E+00
APOE TX (sqrt & std):pos	Endothelial	281	4	1.00E+00	1.00E+00	0	-	-
APOE TX (sqrt & std):pos	Microglia	281	3	1.00E+00	1.00E+00	0	-	-
Total tau TBS (sqrt & std):pos	Microglia	448	7	1.00E+00	1.00E+00	0	-	-
Total tau TX (sqrt & std):pos	Microglia	395	4	1.00E+00	1.00E+00	1	8.87E-01	1.00E+00
p-Tau FA (ln & std):neg	Microglia	607	10	1.00E+00	1.00E+00	0	-	-
p-Tau FA (ln & std):neg	Oligodendrocyte	607	10	1.00E+00	1.00E+00	0	-	-
APOE TX (sqrt & std):pos	Astrocyte	281	1	1.00E+00	1.00E+00	0	-	-
p-Tau FA (ln & std):neg	Endothelial	607	9	1.00E+00	1.00E+00	0	-	-
Total tau TBS (sqrt & std):pos	Endothelial	448	4	1.00E+00	1.00E+00	0	-	-
Total tau TBS (sqrt & std):pos	Astrocyte	448	2	1.00E+00	1.00E+00	0	-	-
Total tau TX (sqrt & std):pos	Astrocyte	395	1	1.00E+00	1.00E+00	0	-	-
Total tau TX (sqrt & std):pos	Endothelial	395	1	1.00E+00	1.00E+00	0	-	-
Total tau TX (sqrt & std):pos	Oligodendrocyte	395	1	1.00E+00	1.00E+00	0	-	-
Total tau TBS (sqrt & std):neg	Neuronal	487	2	1.00E+00	1.00E+00	0	-	-
p-Tau FA (ln & std):pos	Oligodendrocyte	792	9	1.00E+00	1.00E+00	1	9.88E-01	1.00E+00
p-Tau FA (ln & std):neg	Astrocyte	607	4	1.00E+00	1.00E+00	0	-	-
Total tau TBS (sqrt & std):neg	Oligodendrocyte	487	1	1.00E+00	1.00E+00	0	-	-
p-Tau TX (ln & std):pos	Oligodendrocyte	881	7	1.00E+00	1.00E+00	2	9.56E-01	1.00E+00
p-Tau FA (ln & std):pos	Neuronal	792	3	1.00E+00	1.00E+00	0	-	-

p-Tau TX (ln & std):neg	Astrocyte	1304	5	1.00E+00	1.00E+00	0	-	-
p-Tau TX (ln & std):neg	Endothelial	1304	12	1.00E+00	1.00E+00	0	-	-
p-Tau TX (ln & std):neg	Microglia	1304	15	1.00E+00	1.00E+00	1	9.99E-01	1.00E+00
p-Tau TX (ln & std):neg	Neuronal	1304	9	1.00E+00	1.00E+00	0	-	-
p-Tau TX (ln & std):pos	Neuronal	881	2	1.00E+00	1.00E+00	0	-	-

Supplementary Table 3- Correlation between cell type proportion estimates and endophenotypes. Cell type proportions were estimated using DSA deconvolution methods with BRETIGEA marker genes and correlated with each biochemical endophenotype via two sided Kendall Rank Correlation.

AB = Amyloid-beta, FA= insoluble formic acid tissue fraction, ln = natural log transformed, p-Tau = phospho-tau, sqrt = square root transformed, std = standardized, TBS = soluble tris-buffered saline tissue fraction, TCX = Temporal Cortex, t-Tau = Total tau; TX= membrane associated Triton-X tissue fraction

**Endophenotype**	**Cell Type**	**estimate**	**statistic**	**p.value**
Total tau TX (sqrt & std)	Neuronal	0.218	6.770	1.29E-11
Total tau TX (sqrt & std)	Endothelial	-0.213	-6.644	3.05E-11
AB42 FA (ln & std)	Neuronal	0.186	5.793	6.91E-09
AB40/42 TX (ln & std)	Neuronal	-0.173	-5.370	7.89E-08
AB40/42 TX (ln & std)	Oligodendrocyte	0.164	5.115	3.14E-07
Total tau TBS (sqrt & std)	Microglia	-0.163	-5.083	3.72E-07
p-Tau TX (ln & std)	Microglia	0.162	5.026	5.02E-07
p-Tau TX (ln & std)	Astrocyte	0.158	4.906	9.28E-07
p-Tau TBS (sqrt & std)	Neuronal	0.153	4.750	2.03E-06
AB42 TX (ln & std)	Neuronal	0.145	4.528	5.96E-06
AB42 TBS (ln & std)	Neuronal	0.143	4.454	8.42E-06
Total tau FA (sqrt & std)	Neuronal	0.140	4.343	1.40E-05
p-Tau TBS (sqrt & std)	Endothelial	-0.139	-4.332	1.48E-05
Total tau TBS (sqrt & std)	Endothelial	-0.136	-4.231	2.32E-05
AB42 FA (ln & std)	Oligodendrocyte	-0.134	-4.163	3.15E-05
AB42 TBS (ln & std)	Oligodendrocyte	-0.129	-4.030	5.59E-05
APOE TX (sqrt & std)	Microglia	-0.130	-4.022	5.76E-05
AB40 TX (ln & std)	Oligodendrocyte	0.128	3.969	7.23E-05
p-Tau FA (ln & std)	Endothelial	0.127	3.948	7.88E-05
APOE TX (sqrt & std)	Oligodendrocyte	0.126	3.918	8.94E-05
APOE TX (sqrt & std)	Neuronal	-0.124	-3.858	1.14E-04
Braak stage	Endothelial	0.139	3.793	1.49E-04
p-Tau TX (ln & std)	Oligodendrocyte	-0.113	-3.508	4.51E-04
Braak stage	Neuronal	-0.128	-3.500	4.64E-04
AB42 FA (ln & std)	Endothelial	-0.112	-3.487	4.89E-04
p-Tau FA (ln & std)	Astrocyte	0.112	3.470	5.20E-04
Total tau TX (sqrt & std)	Oligodendrocyte	-0.106	-3.287	1.01E-03
AB40/42 TX (ln & std)	Microglia	-0.099	-3.093	1.98E-03
Total tau TBS (sqrt & std)	Astrocyte	-0.097	-3.028	2.47E-03
Thal phase	Astrocyte	0.113	2.997	2.72E-03
p-Tau TBS (sqrt & std)	Astrocyte	0.096	2.975	2.93E-03
AB40 TX (ln & std)	Neuronal	-0.092	-2.854	4.31E-03
p-Tau TBS (sqrt & std)	Oligodendrocyte	-0.090	-2.810	4.95E-03
AB42 TX (ln & std)	Oligodendrocyte	-0.089	-2.781	5.42E-03
Total tau FA (sqrt & std)	Endothelial	-0.087	-2.688	7.19E-03

p-Tau FA (ln & std)	Neuronal	-0.086	-2.682	7.32E-03
AB40 TX (ln & std)	Microglia	-0.082	-2.560	1.05E-02
AB42 TX (ln & std)	Endothelial	-0.080	-2.481	1.31E-02
Total tau FA (sqrt & std)	Oligodendrocyte	-0.079	-2.451	1.42E-02
p-Tau FA (ln & std)	Microglia	0.077	2.406	1.61E-02
Total tau TBS (sqrt & std)	Neuronal	0.077	2.403	1.62E-02
Thal phase	Neuronal	-0.088	-2.357	1.84E-02
sqrt(Avg CAA)	Neuronal	-0.067	-2.097	3.60E-02
AB40/42 FA (ln & std)	Endothelial	0.062	1.917	5.53E-02
Thal phase	Endothelial	0.072	1.906	5.66E-02
APOE TX (sqrt & std)	Astrocyte	-0.061	-1.893	5.83E-02
AB42 TBS (ln & std)	Astrocyte	0.060	1.862	6.26E-02
AB40 FA (ln & std)	Astrocyte	0.058	1.803	7.14E-02
AB42 TBS (ln & std)	Endothelial	-0.058	-1.801	7.17E-02
Total tau FA (sqrt & std)	Microglia	0.057	1.781	7.49E-02
Total tau TBS (sqrt & std)	Oligodendrocyte	0.057	1.776	7.57E-02
AB40/42 FA (ln & std)	Neuronal	-0.057	-1.773	7.62E-02
AB40/42 TX (ln & std)	Endothelial	0.057	1.768	7.71E-02
AB42 TBS (ln & std)	Microglia	0.057	1.760	7.84E-02
AB42 FA (ln & std)	Astrocyte	0.054	1.686	9.17E-02
APOE TBS (sqrt & std)	Oligodendrocyte	-0.053	-1.642	1.01E-01
sqrt(Avg CAA)	Astrocyte	0.051	1.593	1.11E-01
AB42 FA (ln & std)	Microglia	0.047	1.477	1.40E-01
APOE TBS (sqrt & std)	Endothelial	-0.047	-1.477	1.40E-01
Total tau TX (sqrt & std)	Microglia	0.045	1.400	1.61E-01
AB40 TBS (ln & std)	Neuronal	0.044	1.376	1.69E-01
p-Tau TX (ln & std)	Endothelial	0.044	1.373	1.70E-01
APOE FA (ln & std)	Astrocyte	0.044	1.368	1.71E-01
APOE FA (ln & std)	Endothelial	0.044	1.358	1.75E-01
AB40/42 FA (ln & std)	Astrocyte	0.043	1.324	1.86E-01
APOE TBS (sqrt & std)	Neuronal	0.037	1.155	2.48E-01
AB40/42 FA (ln & std)	Oligodendrocyte	0.037	1.151	2.50E-01
AB40 FA (ln & std)	Microglia	0.035	1.087	2.77E-01
APOE TX (sqrt & std)	Endothelial	0.035	1.077	2.81E-01
AB42 TX (ln & std)	Astrocyte	0.033	1.040	2.98E-01
Thal phase	Microglia	0.034	0.915	3.60E-01
sqrt(Avg CAA)	Endothelial	0.029	0.895	3.71E-01
APOE FA (ln & std)	Neuronal	-0.028	-0.876	3.81E-01
AB40 FA (ln & std)	Endothelial	0.027	0.838	4.02E-01
Braak stage	Oligodendrocyte	-0.030	-0.828	4.07E-01
p-Tau FA (ln & std)	Oligodendrocyte	-0.026	-0.803	4.22E-01
AB42 TX (ln & std)	Microglia	0.025	0.792	4.28E-01
p-Tau TBS (sqrt & std)	Microglia	-0.025	-0.788	4.30E-01
AB40/42 TBS (ln & std)	Endothelial	0.025	0.773	4.39E-01
AB40/42 TBS (ln & std)	Oligodendrocyte	0.023	0.726	4.68E-01
p-Tau TX (ln & std)	Neuronal	0.023	0.722	4.70E-01
APOE TBS (sqrt & std)	Astrocyte	-0.022	-0.675	5.00E-01

AB40 TBS (ln & std)	Oligodendrocyte	-0.020	-0.609	5.42E-01
AB40/42 FA (ln & std)	Microglia	0.019	0.582	5.61E-01
AB40/42 TBS (ln & std)	Microglia	-0.018	-0.565	5.72E-01
sqrt(Avg CAA)	Microglia	0.018	0.556	5.78E-01
AB40/42 TX (ln & std)	Astrocyte	-0.018	-0.555	5.79E-01
Total tau FA (sqrt & std)	Astrocyte	0.017	0.541	5.88E-01
Total tau TX (sqrt & std)	Astrocyte	-0.016	-0.488	6.26E-01
AB40 TX (ln & std)	Astrocyte	-0.014	-0.424	6.72E-01
sqrt(Avg CAA)	Oligodendrocyte	0.014	0.422	6.73E-01
Braak stage	Microglia	0.013	0.355	7.22E-01
APOE TBS (sqrt & std)	Microglia	0.011	0.339	7.34E-01
Thal phase	Oligodendrocyte	-0.010	-0.263	7.93E-01
AB40/42 TBS (ln & std)	Neuronal	-0.007	-0.207	8.36E-01
APOE FA (ln & std)	Oligodendrocyte	0.005	0.160	8.73E-01
AB40 FA (ln & std)	Neuronal	0.005	0.145	8.85E-01
AB40 TBS (ln & std)	Astrocyte	0.004	0.136	8.92E-01
AB40 TBS (ln & std)	Microglia	0.003	0.104	9.17E-01
AB40 TX (ln & std)	Endothelial	0.003	0.080	9.36E-01
Braak stage	Astrocyte	0.002	0.063	9.50E-01
AB40 TBS (ln & std)	Endothelial	0.002	0.051	9.60E-01
APOE FA (ln & std)	Microglia	0.001	0.026	9.80E-01
AB40/42 TBS (ln & std)	Astrocyte	0.001	0.019	9.85E-01
AB40 FA (ln & std)	Oligodendrocyte	-0.001	-0.017	9.87E-01

Supplementary Table 4- Number of FDR significant *cis* -eQTM associations by annotation.

Enh = Enhancer, TSS = Transcription start site, ZNF = Zinc finger

rCpGm Annotation		Number of cis-rCpGm-gene pairs
1	Active TSS	1796
2	Flasking TSS	1878
3	Transcription at 5' and 3'	161
4	Strong Transcription	648
5	Weak Transcription	1869
6	Genic enhancers	763
7	Enhancers	3006
8	ZNF genes & repeats	46
9	Heterochromatin	54
10	Bivalent/poised TSS	243
11	Flanking bivalent TSS/Enh	128
12	Bivalent enhancer	187
13	Repressed Polycomb	487
14	Weak repressed Polycomb	658
15	Quiescent/low	995
16	Not Defined	54
Total		**12973**

Supplementary Table 5: FDR Significant TCX mQTL results where the SNP was also significant in an AD-endophenotype GWAS. SNP dosages were tested for association with nearby rCpGms (+/-500,000bp) in a linear regression model adjusting for Age at death, sex, and the first 3 genetic principle components. Significant *cis*- mQTL SNPs were then investigated to determine if the SNP was also significant in our previously published GWAS (Oatman 2023) for each AD endophenotype. *cis* -mQTL-GWAS pairs that were FDR significant (<= 0.05) in the mQTL and GWAS models are shown below. rCpGm ID's are their Chromosome:Start Position:End Position:Chromatin State Annotation of the region with measured CpG sites, not the entire predicted chromatin state region. snpID's are the chromosome:position:A1 allele:A2 allele. Chromatin Annotations defined as '16_NA' are those regions with measured CpGm in hg38, but do not have a corresponsing hg19 position. Confidence interval is 95%. rCpGm positional information is from hg38. A bp distance between rCpGm and SNP of 0 means that the SNP is in the rCpGm window.

A1 = tested allele, A2= non-tested allele, AB = Amyloid-beta, bp = Base pair, conf = confidence interval, Enh = Enhancer, EnhBiv = bivalent enhancer, FA = insoluble formic acid tissue fraction, ln = natural log transformed, N = Number, Quies = Quiescent/low, ReprPC = repressed polycomb, ReprRCWk = weak repressed polycomb, sqrt = square root transformed, TBS = soluble tris-buffered saline tissue fraction, TCX=temporal cortex, TSS = Transcription start site, TssAFlnk = flanking active transcription start site, TssBiv = Bivalent transcription start site, TX = membrane associated Triton-X tissue fraction, ZNF = Zinc finger genes & repeats

				mQTL results					GWAS linear regression results								
rCpGm ID	snpID.hg38	snpID.hg19	avsnp142	estimate	t-stat	pvalue	fdr.pvalue	bp distance between SNP and rCpGm	Pheno	N	estimate	std.error	low.conf	high.conf	pvalue	fdr.pvalue	Gene Closest
chr19:44891929:44892041:2_TssAFlnk	19:44887076:A:G	19:45390333:A:G	rs283815	0.011617686	3.791811736	1.70E-04	2.29E-02	4853	Ab40_42_FA_ratio_ln	441	0.805234	0.117167	0.57559	1.03488	2.20E-11	1.06E-05	*NECTIN2*
chr19:44891929:44892041:2_TssAFlnk	19:44891712:T:G	19:45394969:T:G	rs184017	0.011567079	3.816344059	1.54E-04	2.11E-02	217	Ab40_42_FA_ratio_ln	441	0.807484	0.116225	0.579688	1.03528	1.37E-11	7.07E-06	*TOMM40*
chr19:44891929:44892041:2_TssAFlnk	19:44892457:T:C	19:45395714:T:C	rs157581	0.011668005	3.851763008	1.34E-04	1.88E-02	-416	Ab40_42_FA_ratio_ln	441	0.81597	0.116117	0.588384	1.04356	8.21E-12	4.93E-06	*TOMM40*
chr19:44891929:44892041:2_TssAFlnk	19:44892962:C:T	19:45396219:C:T	rs157582	0.011117984	3.676814482	2.65E-04	3.32E-02	-921	Ab40_42_FA_ratio_ln	441	0.817821	0.11561	0.59123	1.04441	6.07E-12	4.93E-06	*TOMM40*
chr19:44891929:44892041:2_TssAFlnk	19:44893408:G:T	19:45396665:G:T	rs59007384	0.011675126	3.89992745	1.11E-04	1.60E-02	-1367	Ab40_42_FA_ratio_ln	441	0.820724	0.114664	0.595986	1.04546	3.53E-12	3.39E-06	*TOMM40*
chr19:44891929:44892041:2_TssAFlnk	19:44917997:G:A	19:45421254:G:A	rs12721046	0.011634556	3.606612243	3.45E-04	4.12E-02	-25956	Ab40_42_FA_ratio_ln	441	0.665992	0.123097	0.424726	0.907258	1.04E-07	2.12E-02	*APOC1*
chr1:247530849:247531159:14_ReprPCWk	1:247552385:G:A	1:247715687:G:A	rs4925559	-0.030759799	-4.566909503	6.41E-06	1.29E-03	-21226	Ab40_FA_ln	441	0.594011	0.114043	0.370491	0.81753	2.94E-07	2.69E-02	*GCSAML*
chr7:101172738:101173488:13_ReprPC	7:101149581:G:A	7:100792862:G:A	rs13226049	0.045874586	9.624177194	4.70E-20	4.81E-17	23157	Ab40_FA_ln	441	-0.574953	0.113356	-0.797127	-0.352779	5.84E-07	4.18E-02	*SERPINE1;AP1S1*
chr7:101174025:101174713:13_ReprPC	7:101149581:G:A	7:100792862:G:A	rs13226049	0.027922551	7.429044792	5.58E-13	3.13E-10	24444	Ab40_FA_ln	441	-0.574953	0.113356	-0.797127	-0.352779	5.84E-07	4.18E-02	*SERPINE1;AP1S1*
chr7:101172707:101172718:10_TssBiv	7:101149581:G:A	7:100792862:G:A	rs13226049	0.022610133	5.394770276	1.11E-07	3.14E-05	23126	Ab40_FA_ln	441	-0.574953	0.113356	-0.797127	-0.352779	5.84E-07	4.18E-02	*SERPINE1;AP1S1*
chr7:101173631:101173687:12_EnhBiv	7:101149581:G:A	7:100792862:G:A	rs13226049	0.022452371	4.247893858	2.63E-05	4.55E-03	24050	Ab40_FA_ln	441	-0.574953	0.113356	-0.797127	-0.352779	5.84E-07	4.18E-02	*SERPINE1;AP1S1*
chr7:101162691:101162708:14_ReprPCWk	7:101149581:G:A	7:100792862:G:A	rs13226049	-0.008868733	-3.853772476	1.33E-04	1.87E-02	13110	Ab40_FA_ln	441	-0.574953	0.113356	-0.797127	-0.352779	5.84E-07	4.18E-02	*SERPINE1;AP1S1*
chr7:101172738:101173488:13_ReprPC	7:101156292:A:G	7:100799573:A:G	rs4727480	0.044444346	9.310845199	5.72E-19	5.41E-16	16446	Ab40_FA_ln	441	-0.576676	0.112696	-0.797555	-0.355797	4.67E-07	3.42E-02	*AP1S1*
chr7:101174025:101174713:13_ReprPC	7:101156292:A:G	7:100799573:A:G	rs4727480	0.027648241	7.381004332	7.70E-13	4.26E-10	17733	Ab40_FA_ln	441	-0.576676	0.112696	-0.797555	-0.355797	4.67E-07	3.42E-02	*AP1S1*
chr7:101172707:101172718:10_TssBiv	7:101156292:A:G	7:100799573:A:G	rs4727480	0.022467942	5.382054363	1.19E-07	3.34E-05	16415	Ab40_FA_ln	441	-0.576676	0.112696	-0.797555	-0.355797	4.67E-07	3.42E-02	*AP1S1*
chr7:101173631:101173687:12_EnhBiv	7:101156292:A:G	7:100799573:A:G	rs4727480	0.021858644	4.148814372	4.00E-05	6.59E-03	17339	Ab40_FA_ln	441	-0.576676	0.112696	-0.797555	-0.355797	4.67E-07	3.42E-02	*AP1S1*
chr7:101162691:101162708:14_ReprPCWk	7:101156292:A:G	7:100799573:A:G	rs4727480	-0.008519997	-3.713140962	2.31E-04	2.96E-02	6399	Ab40_FA_ln	441	-0.576676	0.112696	-0.797555	-0.355797	4.67E-07	3.42E-02	*AP1S1*
chr7:101172738:101173488:13_ReprPC	7:101157024:C:T	7:100800305:C:T	rs35811390	0.044595954	9.328065528	4.99E-19	4.74E-16	15714	Ab40_FA_ln	441	-0.575442	0.112911	-0.796744	-0.354141	5.18E-07	3.74E-02	*AP1S1*
chr7:101174025:101174713:13_ReprPC	7:101157024:C:T	7:100800305:C:T	rs35811390	0.027853529	7.427041025	5.66E-13	3.17E-10	17001	Ab40_FA_ln	441	-0.575442	0.112911	-0.796744	-0.354141	5.18E-07	3.74E-02	*AP1S1*
chr7:101172707:101172718:10_TssBiv	7:101157024:C:T	7:100800305:C:T	rs35811390	0.022407771	5.356114269	1.36E-07	3.79E-05	15683	Ab40_FA_ln	441	-0.575442	0.112911	-0.796744	-0.354141	5.18E-07	3.74E-02	*AP1S1*
chr7:101173631:101173687:12_EnhBiv	7:101157024:C:T	7:100800305:C:T	rs35811390	0.022056631	4.179781375	3.51E-05	5.87E-03	16607	Ab40_FA_ln	441	-0.575442	0.112911	-0.796744	-0.354141	5.18E-07	3.74E-02	*AP1S1*
chr7:101162691:101162708:14_ReprPCWk	7:101157024:C:T	7:100800305:C:T	rs35811390	-0.008628532	-3.754724814	1.96E-04	2.59E-02	5667	Ab40_FA_ln	441	-0.575442	0.112911	-0.796744	-0.354141	5.18E-07	3.74E-02	*AP1S1*
chr8:134326024:134401840:15_Quies	8:134330581:A:C	8:135342824:A:C	rs13255060	-0.025367556	-7.213498234	2.34E-12	1.23E-09	0	Ab40_FA_ln	441	0.70369	0.133757	0.441531	0.965848	2.26E-07	2.69E-02	*ST3GAL1;ZFAT*
chr19:44891929:44892041:2_TssAFlnk	19:44887076:A:G	19:45390333:A:G	rs283815	0.011617686	3.791811736	1.70E-04	2.29E-02	4853	Ab40_FA_ln	441	0.761275	0.121919	0.522319	1.00023	1.02E-09	5.26E-04	*NECTIN2*
chr19:44891929:44892041:2_TssAFlnk	19:44891712:T:G	19:45394969:T:G	rs184017	0.011567079	3.816344059	1.54E-04	2.11E-02	217	Ab40_FA_ln	441	0.765369	0.120932	0.528346	1.00239	6.16E-10	3.45E-04	*TOMM40*
chr19:44891929:44892041:2_TssAFlnk	19:44892457:T:C	19:45395714:T:C	rs157581	0.011668005	3.851763008	1.34E-04	1.88E-02	-416	Ab40_FA_ln	441	0.767531	0.12093	0.530512	1.00455	5.54E-10	3.39E-04	*TOMM40*
chr19:44891929:44892041:2_TssAFlnk	19:44892962:C:T	19:45396219:C:T	rs157582	0.011117984	3.676814482	2.65E-04	3.32E-02	-921	Ab40_FA_ln	441	0.767271	0.120447	0.5312	1.00334	4.82E-10	3.24E-04	*TOMM40*
chr19:44891929:44892041:2_TssAFlnk	19:44893408:G:T	19:45396665:G:T	rs59007384	0.011675126	3.89992745	1.11E-04	1.60E-02	-1367	Ab40_FA_ln	441	0.785487	0.119257	0.551749	1.01923	1.30E-10	1.46E-04	*TOMM40*
chr19:44891929:44892041:2_TssAFlnk	19:44893408:G:T	19:45396665:G:T	rs59007384	0.011675126	3.89992745	1.11E-04	1.60E-02	-1367	Ab40_TX_ln	441	0.311029	0.0588921	0.195603	0.426456	2.03E-07	4.27E-02	*TOMM40*
chr19:44891929:44892041:2_TssAFlnk	19:44887076:A:G	19:45390333:A:G	rs283815	0.011617686	3.791811736	1.70E-04	2.29E-02	4853	apoE_FA_ln	441	0.424088	0.0597025	0.307073	0.541103	5.02E-12	1.99E-06	*NECTIN2*
chr19:44891929:44892041:2_TssAFlnk	19:44891712:T:G	19:45394969:T:G	rs184017	0.011567079	3.816344059	1.54E-04	2.11E-02	217	apoE_FA_ln	441	0.422723	0.05926	0.306575	0.53887	4.13E-12	1.85E-06	*TOMM40*
chr19:44891929:44892041:2_TssAFlnk	19:44892457:T:C	19:45395714:T:C	rs157581	0.011668005	3.851763008	1.34E-04	1.88E-02	-416	apoE_FA_ln	441	0.422864	0.0592727	0.306692	0.539037	4.11E-12	1.85E-06	*TOMM40*
chr19:44891929:44892041:2_TssAFlnk	19:44892962:C:T	19:45396219:C:T	rs157582	0.011117984	3.676814482	2.65E-04	3.32E-02	-921	apoE_FA_ln	441	0.420298	0.0590705	0.304521	0.536074	4.65E-12	1.95E-06	*TOMM40*
chr19:44891929:44892041:2_TssAFlnk	19:44893408:G:T	19:45396665:G:T	rs59007384	0.011675126	3.89992745	1.11E-04	1.60E-02	-1367	apoE_FA_ln	441	0.425004	0.058533	0.310282	0.539727	1.79E-12	1.34E-06	*TOMM40*
chr19:44891929:44892041:2_TssAFlnk	19:44917997:G:A	19:45421254:G:A	rs12721046	0.011634556	3.606612243	3.45E-04	4.12E-02	-25956	apoE_FA_ln	441	0.390246	0.0622648	0.268209	0.512283	8.86E-10	2.48E-04	*APOC1*
chr19:44891929:44892041:2_TssAFlnk	19:44887076:A:G	19:45390333:A:G	rs283815	0.011617686	3.791811736	1.70E-04	2.29E-02	4853	apoE_TBS_sqrt	441	-2.41065	0.352174	-3.1009	-1.7204	2.62E-11	7.71E-05	*NECTIN2*
chr19:44891929:44892041:2_TssAFlnk	19:44891712:T:G	19:45394969:T:G	rs184017	0.011567079	3.816344059	1.54E-04	2.11E-02	217	apoE_TBS_sqrt	441	-2.38015	0.349933	-3.066	-1.69429	3.44E-11	7.71E-05	*TOMM40*
chr19:44891929:44892041:2_TssAFlnk	19:44892457:T:C	19:45395714:T:C	rs157581	0.011668005	3.851763008	1.34E-04	1.88E-02	-416	apoE_TBS_sqrt	441	-2.38457	0.349951	-3.07046	-1.69868	3.18E-11	7.71E-05	*TOMM40*
chr19:44891929:44892041:2_TssAFlnk	19:44892962:C:T	19:45396219:C:T	rs157582	0.011117984	3.676814482	2.65E-04	3.32E-02	-921	apoE_TBS_sqrt	441	-2.34162	0.349192	-3.02602	-1.65721	6.26E-11	8.42E-05	*TOMM40*
chr19:44891929:44892041:2_TssAFlnk	19:44893408:G:T	19:45396665:G:T	rs59007384	0.011675126	3.89992745	1.11E-04	1.60E-02	-1367	apoE_TBS_sqrt	441	-2.26983	0.347608	-2.95113	-1.58854	1.84E-10	1.77E-04	*TOMM40*
